# Multi‐Pulse Corona Discharges in Thunderclouds Observed in Optical and Radio Bands

**DOI:** 10.1029/2022GL098938

**Published:** 2022-07-08

**Authors:** Dongshuai Li, Alejandro Luque, Nikolai G. Lehtinen, F. J. Gordillo‐Vázquez, Torsten Neubert, Gaopeng Lu, Olivier Chanrion, Hongbo Zhang, Nikolai Østgaard, Víctor Reglero

**Affiliations:** ^1^ Instituto de Astrofísica de Andalucía (IAA) CSIC Granada Spain; ^2^ Now at National Space Institute Technical University of Denmark (DTU Space) Kongens Lyngby Denmark; ^3^ Birkeland Centre for Space Science, Department of Physics and Technology University of Bergen Bergen Norway; ^4^ National Space Institute Technical University of Denmark (DTU Space) Kongens Lyngby Denmark; ^5^ CAS Key Laboratory of Geospace Environment University of Science and Technology of China Hefei China; ^6^ Key Laboratory of Middle Atmosphere and Global Environment Observation (LAGEO) Institute of Atmospheric Science Chinese Academy of Sciences Beijing China; ^7^ Image Processing Laboratory University of Valencia Valencia Spain

## Abstract

How lightning initiates inside thunderclouds remains a major puzzle of atmospheric electricity. By monitoring optical emissions from thunderstorms, the Atmosphere‐Space Interactions Monitor (ASIM) onboard the International Space Station is providing new clues about lightning initiation by detecting Blue LUminous Events (BLUEs), which are manifestations of electrical corona discharges that sometimes precedes lightning. Here we combine optical and radio observations from a thunderstorm near Malaysia to uncover a new type of event containing multiple optical and radio pulses. We find that the first optical pulse coincides with a strong radio signal in the form of a Narrow Bipolar Event (NBE) but subsequent optical pulses, delayed some milliseconds, have weaker radio signals, possibly because they emanate from a horizontally oriented electrical discharges which does not trigger full‐fledged lightning. Our results cast light on the differences between isolated and lightning‐initiating electrical discharges.

## Key Points

- Optical multi‐pulse corona discharges coincide with Narrow Bipolar Events and their subsequent pulses
- Subsequent optical pulses are related to horizontally oriented electrical discharges which sometimes emit weak radio signals
- The class of horizontally oriented electrical discharges might play a significant role in the initiation of the lightning leaders

## 1 Introduction

Narrow bipolar events (NBEs) are short (10–20 µs) radio pulses emitted by thunderclouds (Le Vine, 1980; Smith et al., 1999, 2004). Their source, hypothesized to be a special electrical discharge process named fast breakdown (Rison et al., 2016; Tilles et al., 2019), has received intense interest in recent years. Fast breakdown is likely present in all lightning initiation events (Attanasio et al., 2021) and even during flash development, although only under a still undefined set of conditions it is sufficiently strong to manifest itself as an NBE. The propagation speed of fast breakdown (10^7^–10^8^ m/s) (Rison et al., 2016) as well as space‐based observations of blue flashes coinciding with NBEs (Li et al., 2021; Soler et al., 2020) suggest that fast breakdown consists in the simultaneous propagation of 10^8^ to 10^9^ (N. Liu et al., 2019) cold filamentary discharges called streamers (Nijdam et al., 2020).

NBEs normally occur in isolation (Kostinskiy et al., 2020; Rison et al., 2016) but a small fraction of them, named Initiation‐type NBEs (INBEs) (Wu et al., 2014) are the initial event of a lightning flash. Sometimes NBEs are followed by subsequent radio pulses associated with leaders (hot lightning channels); these pulses, called Initial Breakdown Pulses (IBPs) (e.g., Kostinskiy et al., 2020; Lyu et al., 2019) or Preliminary Breakdown pulses (PBs) (e.g., Kolmašová et al., 2018; Wu et al., 2015), last for a few milliseconds at the initial stage of an intracloud (IC) or cloud‐to‐ground (CG) lightning discharge. Whereas isolated NBEs are strong emitters of Very High Frequency (VHF) radiation (3,000−300,000 W) (Kostinskiy et al., 2020; Rison et al., 2016), initiation‐type NBEs, even with pulse widths similar to isolated NBEs, present smaller amplitudes and weaker VHF signals (3–300 W) (Bandara et al., 2019; Kostinskiy et al., 2020; Rison et al., 2016; Wu et al., 2014).

Most of this knowledge about NBEs and fast breakdown derives from radio observations but these have been recently complemented by optical detections from space. The Modular Multispectral Imaging Array (MMIA) instrument of the Atmosphere‐Space Interactions Monitor (ASIM), operating since 2018 from the International Space Station (ISS), has detected a large number of Blue LUminous Events (BLUEs) globally (Soler et al., 2021), which are optical pulses with a strong 337 nm signal, associated with streamer discharges, but lacking the 777 nm emissions that would indicate the presence of a hot leader (Li et al., 2021; F. Liu, Lu, et al., 2021; Soler et al., 2020). Combined radio and optical studies have found that NBEs often have a BLUE counterpart (Li et al., 2021; F. Liu, Lu, et al., 2021; Soler et al., 2020).

Novel optical observations help to elucidate the context in which NBEs occur. Soler et al. (2020) found that a significative fraction of BLUEs contained more than one optical pulse with a delay between pulses of a few milliseconds. Here we combine observations from MMIA and from ground‐based Very Low Frequency/Low Frequency (VLF/LF) radio sensors to investigate a number of multi‐pulse BLUEs inside a thunderstorm near Malaysia. We find that in all these events the first optical pulse has an unambiguous NBE counterpart. Remarkably, we also find that the subsequent optical pulses, even though they have optical amplitudes comparable to the leading pulse, are either accompanied by weaker radio emissions or do not even have a radio counterpart that can be discerned over the noise. The implication is that NBEs might be followed by a new type of event that has escaped detection until now. Our observations are compatible with these events being horizontally directed electrical discharges which does not initiate a leader.

## 2 Instruments and Observations

Since its commissioning in 2018, the Modular Multispectral Imaging Array (MMIA) of the Atmosphere‐Space Interactions Monitor (ASIM) has been observing Earth thunderstorms from space in a nadir‐viewing geometry from the International Space Station (ISS) (Chanrion et al., 2019; Neubert et al., 2019). MMIA contains three photometers with a sampling rate of 100 k samples/s: one photometer in the ultraviolet (UV) band at 180–230 nm, one in the near‐UV at the strongest spectral line of the nitrogen second positive system (337 nm) and one at the strongest lightning emission band (777.4 nm). The last two photometers are complemented with cameras sensitive to the same wavelengths. The spatial resolution of the cameras on the ground is around 400 × 400 m with an integration time of 83.3 ms.

Our radio‐frequency data comes from a broadband Very Low Frequency/Low Frequency (VLF/LF) magnetic sensor that operates at 400 Hz to 400 kHz and is located at Universiti Teknikal Malaysia Melaka (UTeM), Malacca, Malaysia (Ahmad et al., 2017; Zhang et al., 2016). To compare MMIA and VLF/LF data correcting for MMIA's time uncertainty we matched MMIA pulses with data from the GLD360 lightning detection network (Said & Murphy, 2016), obtaining a time shift for MMIA with respect to the ground‐based VLF/LF measurements of (−15.00 ± 0.65) ms (see Figure S1 in Supporting Information S1).

On the evening of 30 April 2020, there were 16 Blue LUminous Events (BLUEs) simultaneously observed by the 337 nm photometer and its filtered camera of MMIA, as well as the ground‐based VLF/LF sensor near Malaysia (Ahmad et al., 2017), with absent or negligible signals in both the 180–230 nm photometer and in the 777.4 nm photometer and filtered camera. Among the events, there are 8 single‐pulse BLUEs (Li et al., 2021; Soler et al., 2020) and 8 multiple‐pulse BLUEs (2 special multiple‐pulse events). We focus mainly on the multiple‐pulse BLUEs.

**Figure 1:**
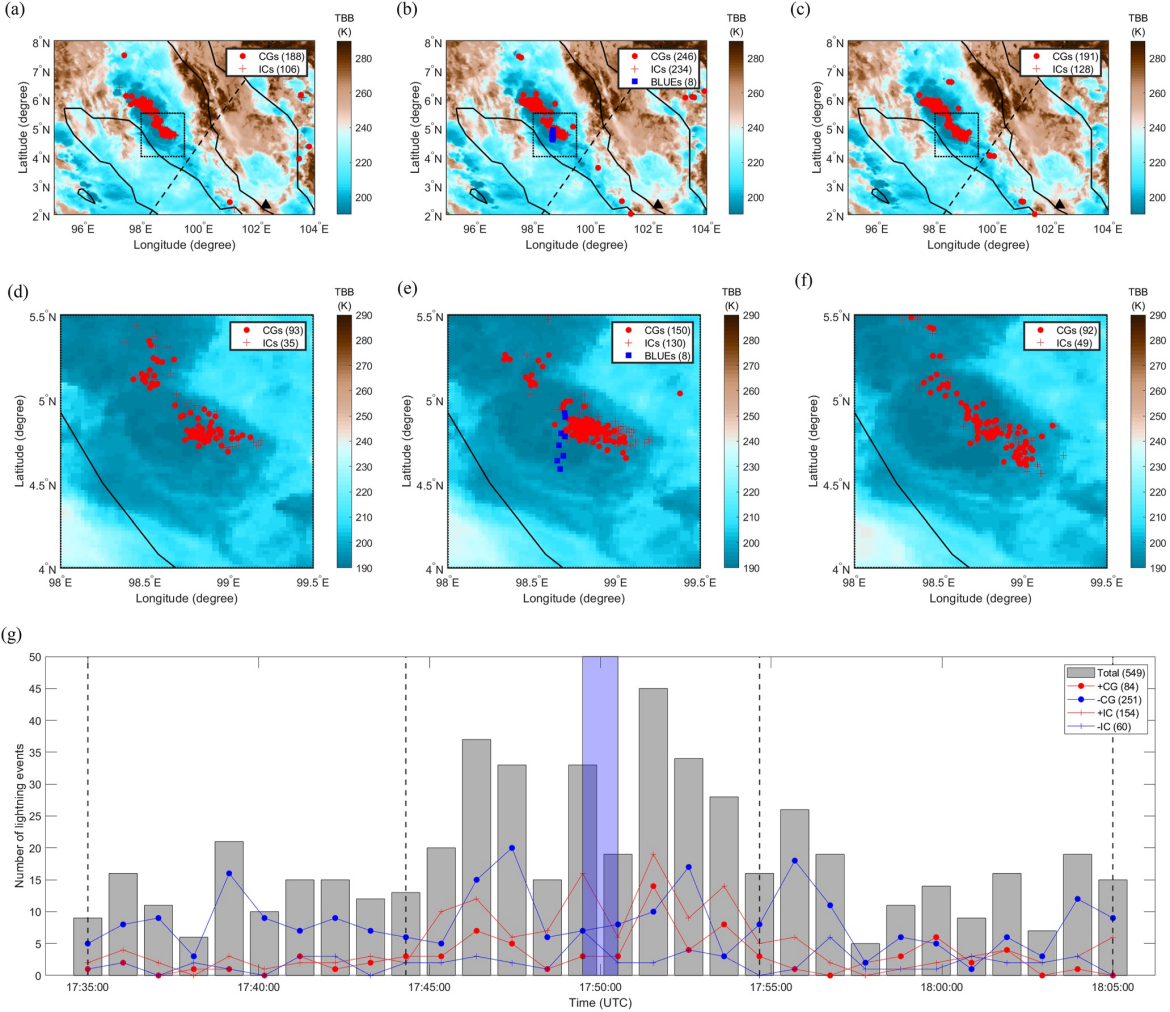
Distribution of the intracloud (IC)/cloud‐to‐ground (CG) lightning with 8 multiple‐pulse BLUEs superimposed on the cloud Top Blackbody Brightness temperature (TBB, given in (K) in the region of interest and the zoomed‐in rectangular region, indicated with the dotted black line, per 10 min at time 17:40:00 UTC (a),(d), 17:50:00 UTC (b),(e), and 18:00:00 UTC (c),(f). Numbers of different types of lightning events are shown in (g): positive CGs (+CGs), negative CGs (−CGs), positive ICs (+ICs) and negative ICs (−ICs). The multiple‐pulse BLUEs occurred in the time period from 17:50:00 to 17:51:00 UTC marked in blue shaded region in (g). The ground‐based VLF/LF sensor at Malaysia is shown as a black triangle in panels (a, b, c). The footprints of ASIM are shown with a black dashed line.

To illustrate the thunderstorm context of the BLUEs, Figure 1 shows the distribution of intracloud (IC)/cloud‐to‐ground (CG) lightning with the eight multi‐pulsed BLUEs superimposed on the cloud Top Blackbody Brightness temperature (TBB, given in Kelvin) provided by the Himawari‐8 satellite (Bessho et al., 2016) in ten‐minute intervals starting at 17:40:00 UTC, 17:50:00 UTC and 18:00:00 UTC. Because GLD360 only captured three events, we determine the locations of multiple‐pulse BLUEs by projecting the brightest pixel of the 337‐nm camera images into geo‐coordinates (latitude and longitude). We also show the GLD360‐detected lightning flashes surrounding our events, including their classification as positive or negative, CG or IC. The BLUEs, which occurred in the time period from 17:50:00 to 17:51:00 UTC, are accompanied by the highest concentration of IC and CG lightning with an apparent decrease of the negative CG flash rate.

**Figure 2:**
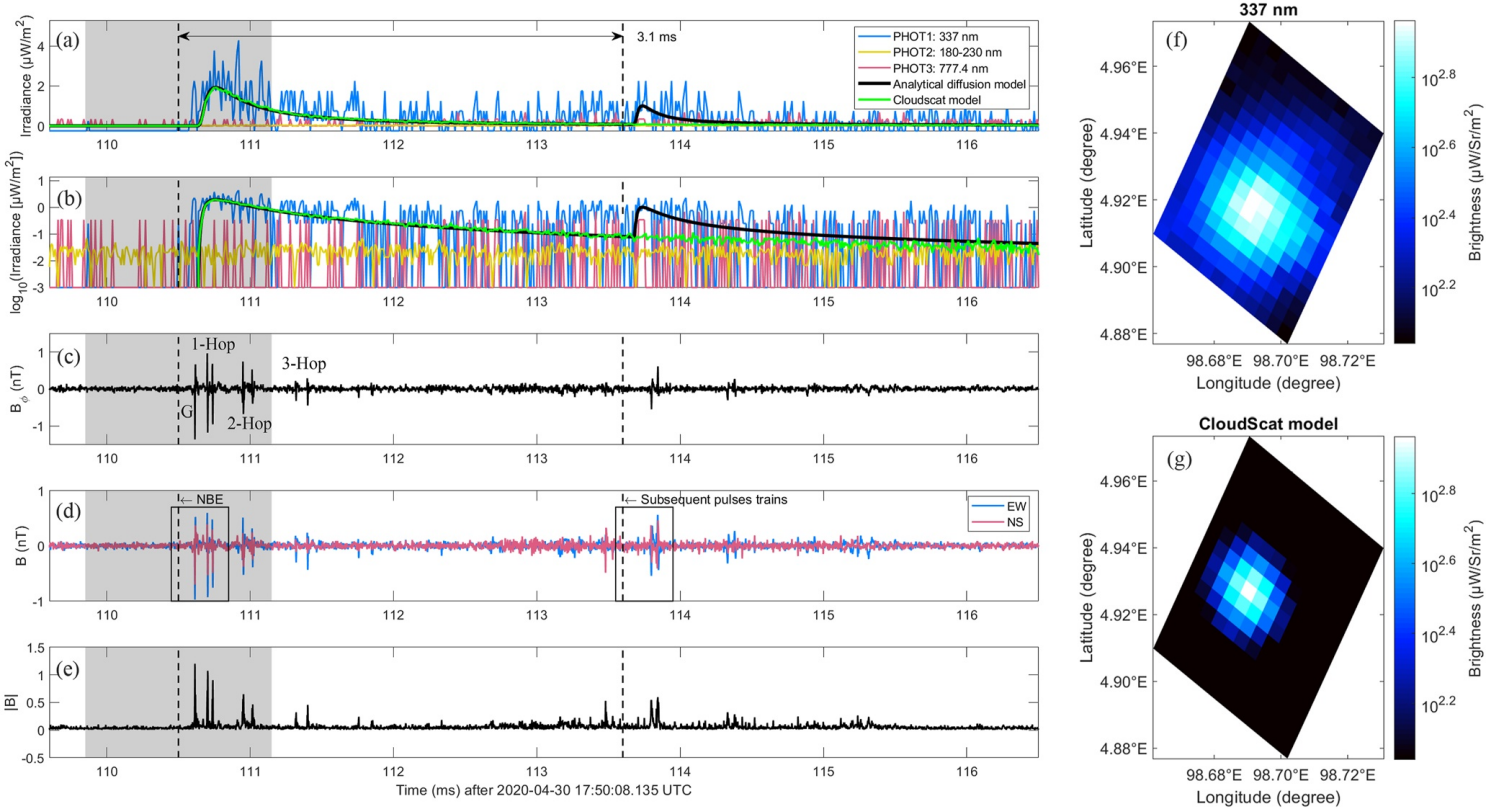
Comparison between MMIA photometer irradiance (blue: 337 nm, yellow: 180–230 nm and red: 777.4 nm) and the modeling results of the analytical diffusion model (black) and Cloudscat model (green) on a linear (a) and logarithmic (b) scale along with azimuthal magnetic field component *B*_*ϕ*_ (c), the North‐south and East‐west magnetic field components *B*_NS_ and *B*_EW_ (d) and its norm $|B|=\sqrt{B_{\mathrm{NS}}^{2}+B_{\mathrm{EW}}^{2}}$ (e), recorded at the ground‐based VLF/LF sensor nearby Malaysia for event 1. Also shown: the image detected by the 337‐nm filtered camera of MMIA (f) and the simulated image of the Cloudscat model (g). The start time (corrected to the source time with respect to the locations) for NBE and its subsequent pulses is marked with the dashed black line, within the time difference 3.1 ms with ±0.65 ms uncertainty (gray shadowed region). The ground wave and multiple‐hop ionospheric reflections for NBEs are marked in (c).

The detailed features of all multiple‐pulse BLUEs are listed in Table 1. As an example, the multiple‐pulse BLUE with ID 1 is presented in Figure 2, with other cases given in Figure S3–S10 in Supporting Information S1. The multiple‐pulse BLUEs include one primary BLUE pulse and one or several subsequent BLUE pulses within 1–9 ms which are identified based on the binned average of 15 data points (150 µs) of the 337‐nm photometer signal (see Figure S2 in Supporting Information S1). Both primary and secondary BLUE pulses are statically significant with their signals above *μ* ± 5*σ* level of the background noise, where *μ* is the empirical average and *σ* is the standard deviation (see Figure S11–S18 in Supporting Information S1).

**Table 1:** The Detailed Features of the Multiple‐Pulse BLUEs

		Primary BLUE			Subsequent BLUE pluses			
ID	MMIA UTC time (source)	Irradiance (μW/m^2^)	Rise time^a^ (ms)	Total time duration^b^ (ms)	Irradiance (μW/m^2^)	Rise time^a^ (ms)	Total time duration^b^ (ms)	Time difference(ms)
1	17:50:08.246	4.54	0.18	1.25	2.5	0.05	0.86	3.1
2	17:50:09.645	5.57	0.30	2.25	2.76^c^	0.58^c^	1.66^c^	6.0
3	17:50:19.447	12.42	0.17	1.53	6.08	0.14	1.61	1.7
4	17:50:24.704	10.28	0.79	6.50	3.52	0.14	3.37	9.4
5	17:50:35.617	4.54	0.59	2.45	4.54	0.68	2.64	3.3
6^*^	17:50:43.238	8.69	0.79	3.60	4.54	0.79	3.75	2.6
7^*^	17:50:46.157	10.81	0.06	1.93	3.01	0.22	1.15	7.3
8	17:50:55.181	3.01	0.12	1.19	3.01^c^	0.41^c^	2.55^c^	1.4

*Note*. The detection times of MMIA have been corrected to the source time with respect to the BLUE locations.

^a^ Rise time is the time taken for the amplitude of a MMIA photometer signal to rise from 10% to 90%.

^b^ Time duration is the time interval for the amplitude of a MMIA photometer signal to rise from 10% and fall to 10%.

^c^ The first subsequent BLUE pulse is used to evaluate the rise time and time duration since the photometer signal includes multiple pulses (see Figure S4 and S10 in Supporting Information S1 for details).

^*^ The special multiple‐pulse case.

In most cases, both rise time and time duration of the subsequent BLUE pulses are found to be similar or somewhat shorter than those corresponding to the primary BLUEs; the irradiances of the primary BLUEs are higher than those of the subsequent BLUE pulses by about a factor of two. All the BLUEs are isolated from other IC or CG lightning discharges detected by either GLD360 or the 777.4 nm photometer and filtered camera of MMIA within at least 100 ms. That means that they do not initiate any leader activity and therefore would be classified as isolated NBEs.

## 3 Results

As shown in Figure 2 and Figures S3–S10 in Supporting Information S1, all the multiple‐pulse BLUEs are associated with positive NBEs. The NBE pulses, including both ground wave (50 µs time scale in Figure S28 in Supporting Information S1) and multiple‐hop ionospheric reflections, are shown in more detail in Figures S19–S27 in Supporting Information S1. The presence of subsequent optical signals with magnitudes comparable to the leading pulse hints to the existence of substantial electrical activity in the thundercloud up to several milliseconds after the initial discharge. However, although in all cases the initial discharge produces a clear NBE signature in the VLF/LF, the subsequent, presumed electrical activity is in many cases not observed in the VLF/LF signal or, if present, is weak. Among all events, four (ID 1, ID 6, ID 7, and ID 8 (after 133 ms)) are accompanied by weak subsequent radio pulses or enhanced radio noise detected by the VLF/LF sensor near Malaysia (see Figures S3, S8, S9, and S10 in Supporting Information S1). For the cases with ID 2, ID 4, and ID 5 there is no radio signal above the background noise (see Figures S4, S6, and S7 in Supporting Information S1). The subsequent pulse trains for cases with ID 3 (after 148 ms) and ID 8 (after 130) are not obvious and might overlap with the multiple‐hop ionospheric pulses (see Figures S5 and S10 in Supporting Information S1). Note that there is a few pulses before the subsequent pulse trains only detected in the North‐south magnetic field component in Figure 2d, not in the azimuthal magnetic field signal (see Figure 2c), which might due to the unrelated sources or random noises measured from the North‐south antenna.

**Figure 3:**
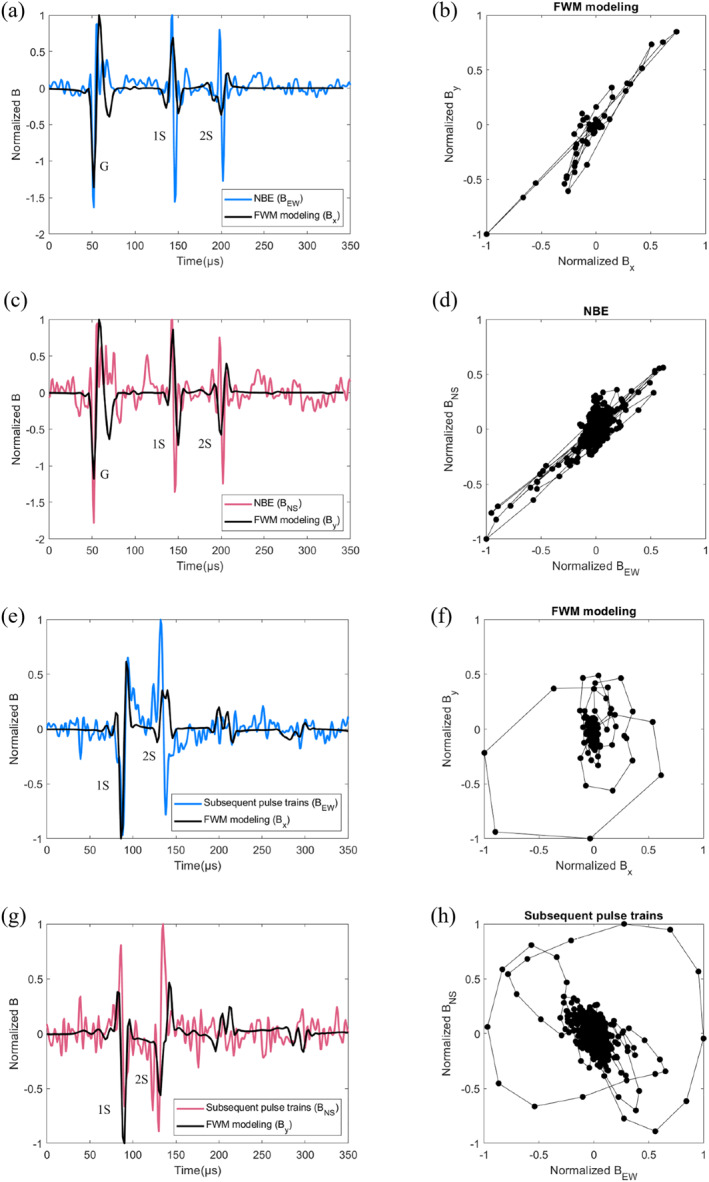
Comparison of normalized magnetic field components between the simulation and observation corresponding to the NBE (a,b,c,d) and the subsequent pulses (e,f,g,h) of multiple‐pulse BLUE for event 1 (see the black rectangle with the NBE and subsequent pulses labels marked in Figure 2(d)). The magnetic field components of *B*_*x*_ and *B*_*y*_ are calculated by the FWM modeling and the East‐West and North‐South magnetic field components of *B*_EW_ and *B*_NS_ are measured by the ground‐based VLF/LF sensor at Malaysia. The correlation between the different components of the simulated magnetic field (*B*_*x*_ and *B*_*y*_) and the measured magnetic field components (*B*_EW_ and *B*_NS_) for both NBE (b), (d) and the subsequent pulses (f), (h) are also shown in the figure. The ground wave and 1‐hop sky wave reflections are marked as G, 1S, and 2S.

Figure 3 further demonstrate the correlation of the horizontal *B* fields (*B*_EW_ and *B*_NS_) for both the positive NBE and its subsequent pulses for event 1 (See Figures S19–S27 in Supporting Information S1 for other cases). The ground wave and the 1‐hop sky wave reflections, including the path directly reflected from the ionosphere and the path first reflected from the surface of the earth and then from the ionosphere, which are marked as G, 1S, and 2S in the radio signals. As shown in Figure 3d, the NBE pulses exhibit a tight linear relationship of the horizontal *B* fields, something that is expected for the horizontally propagating ground wave of a vertical discharge. However, in the subsequent pulses the horizontal components of the field trace elliptical curves (see Figure 3h). As this is a projection into the horizontal plane of the trajectory of the magnetic field in the plane perpendicular to the wave propagation, the implication is that the wave is elliptically polarized. One explanation for this behavior is that the electric current responsible for the subsequent pulses is oriented horizontally: in that case the ground wave is absent and the first signal to reach the detector is the wave reflected in the ionosphere. Due to the anisotropy introduced by the geomagnetic field, different components of the wave electromagnetic fields propagate differently in the magnetized plasma of the lower ionosphere, introducing a phase shift between different components. This would explain both the weak amplitude and the elliptical polarization of the observed signal.

We tested this hypothesis by means of a Full Wave Method (FWM) electromagnetic model (Lehtinen & Inan, 2008, 2009) by considering the ionosphere as a magnetized plasma (Alken et al., 2021; Bilitza et al., 2014) (see Methodology in Supporting Information S1 for further details). We simulated the signals produced by both vertical and horizontal dipole current sources imitating the event‐detector geometry of our observations. The results, shown in Figure 3, reproduce the general features of both ground wave and 1‐hop sky wave reflections radiated from the vertically and horizontally oriented sources. Note that there are unmatched features between the observed and simulated radio signals in Figure 3 which may be partly due to the direction of the horizontal discharges, the frequency limitation of the FWM model (10–100 kHz) (Lehtinen & Inan, 2008, 2009) as well as imperfectly accounting for the effect of the ground and the ionospheric cold plasma characteristics (Li et al., 2019, 2020).

For the case of NBE, both ground wave and its 1‐hop sky wave reflections can be clearly seen in Figures 3a and 3c with a linear relationship between the magnetic field components *B*_*x*_ and *B*_*y*_ (see Figures 3b and 3d). For the subsequent pulse trains, the ground wave is absent and the magnetic field components *B*_*x*_ and *B*_*y*_ of the 1‐hop sky wave reflections trace elliptical curves (see Figure 3e,f,g,h). These simulations support our hypothesis of a horizontal discharge triggered by the primary, vertical breakdown that generates the NBE. Note that event 8 is another case showing a clear image of elliptical polarization to support our hypothesis (see Figure S27 in Supporting Information S1).

To better understand the multiple‐pulse BLUEs, we simulated the propagation of their optical emissions within the thundercloud by using both an analytical diffusion model (Koshak et al., 1994; Soler et al., 2020; Wilkman, 2013) and a Monte Carlo model (Luque et al., 2020) (see Methodology in Supporting Information S1 for further details). We only fit the BLUEs with a clear impulsive pulse and considered a fit as good when the coefficient of determination *R* ^2^ > 0.6 (Chicco et al., 2021). The analytical diffusion model (black solid line in Figure 2) is based on Equation (2) in Supporting Information S1 to simulate both primary and secondary optical pulses. Table 2 lists the inferred parameters of the multiple‐pulse BLUEs. The estimated depths *L* (relative to the cloud top) of the BLUEs are derived by the analytical diffusion model based on the 337‐nm photometer signals of MMIA, assuming a cloud particle radius *r* = 20 μm and droplet number density *N*_*d*_ = 1 × 10^8^ m^−3^ to 3 × 10^8^ m^−3^. The altitudes *H* of the NBEs are inferred from the propagation distance and the difference in times of arrival from the direct ground wave and 1‐hop sky wave reflections in the ground‐based VLF/LF radio signals by using the simplified ray‐theory method (Smith et al., 1999, 2004), which involves an uncertainty of about ±1 km when compared with the full‐wave results (Li et al., 2020).

**Table 2:** The Inferred Features of the Multiple‐Pulse BLUEs

	Parameters			NBE					Subsequent pulses
ID	Distance *d* (km)	*N*_*d*_ (m^−3^)	*R* (μm)	337‐nm optical energy (J)	Streamer branching events	Altitude *H* (km)	Depth *L* (km)	Current moment *M*_*i*_ (kA ⋅ km)	Depth *L* (km)
1	495	3 × 10^8^	20	1.3 × 10^3^	1.0 × 10^8^	17.68	0.96	2.64	0.66
2	494	2 × 10^8^	20	3.7 × 10^3^	2.9 × 10^8^	16.67	1.50	5.50	1.74^a^
3	489	1 × 10^8^	20	6.4 × 10^3^	5.0 × 10^8^	17.03	1.85	4.21	1.45
4^b^	486	–	–	–	–	15.55	–	6.23	–
5^b^	486	–	–	–	–	15.55	–	2.92	–
6^b^ ^*^	480	–	–	–	–	15.87	–	3.69	–
7^*^	482	1 × 10^8^	20	3.8 × 10^3^	3.0 × 10^8^	17.95	1.30	22.62	2.79
8^c^	477	–	–	–	–	17.33	–	3.64	–

*Note*. The altitudes (*H*) are estimated using the simplified ray‐theory method proposed by (Smith et al., 1999, 2004) based on the ground‐based VLF/LF sferics. The depths (*L*) relative to cloud top boundary are evaluated by using the analytical diffusion model in equation (2) in Supporting Information S1 based on the 337‐nm photometer signals of MMIA.

^a^ The first subsequent BLUE pulse is used to obtain the fitting parameters since the photometer signal includes multiple subsequent BLUE pulses (see Figure S4 in Supporting Information S1 for details).

^b^ There is a small pulse on the rising edge of light‐curve that distorted the fit process (See Figure S6, S7 and S8 in Supporting Information S1 for details).

^c^ The photometer signal is too noisy to be fitted (See Figure S10 in Supporting Information S1 for details).

^*^ The special multiple‐pulse case.

We see in Table 2 that the positive NBEs are located at relatively high altitudes with *H* = 16–18 km, which are above the median heights of the majority of positive NBEs (about 13 km) reported in the literature (F. Liu, Zhu, et al., 2021; Smith et al., 2004; Wu et al., 2014). This suggests that the occurrence of multi‐pulsed BLUE events may be related to the rare occurrence of high‐altitude positive NBEs (Wu et al., 2014). As shown in Table 2, our modeling results indicate that the subsequent BLUE pulses are located at similar or slightly higher altitudes than the primary BLUEs, with a depth of *L* = 1–3 km measured from the cloud top. This low depth explains why MMIA detects not only the primary BLUEs but also their subsequent BLUE pluses. The optical energy in the 337‐nm band emitted by the breakdown of the primary NBE is about 10^3^ J, which involves around 10^8^ streamer branching events evaluated as discussed by Li et al. (2021). The ratio of the irradiances and the streamer branches is expected to have a roughly linear relationship, thus the secondary BLUEs involve about 5 × 10^7^ streamer breaching events.

We can shed some light into what differentiates multiple‐pulse from single‐pulse BLUEs by looking at the electrical currents of the breakdowns where they originate. We estimated the current moments (*M*_*i*_) of all the multiple‐pulse BLUEs (see Table 2) with sufficient data in the investigated thunderstorm, including the 8 multiple‐pulse BLUEs (2 special multiple‐pulse events) and the 8 single‐pulse BLUEs. Starting from the azimuthal magnetic field component, *B*_*ϕ*_, we solved the inverse convolution problem (Cummer, 2003; Cummer & Inan, 2000) using the Uman's equation (Uman et al., 1975) based on the ground wave signals measured by the ground‐based VLF/LF sensor at Malaysia (see Methodology and Figure S28 and S29 in Supporting Information S1 for further details). Figure S30 in Supporting Information S1 shows the amplitude of the azimuthal magnetic field component *B*_*ϕ*_ and the estimated current moment *M*_*i*_ (which are obviously linearly correlated). The two special multiple‐pulse events are marked in green dots in Figure S30 in Supporting Information S1 since the subsequent pulse trains of them seem to be a “NBE‐like” event, which might two NBE events occurred closely in time (see Figures S24e and S24f and Figures S25e and S25f in Supporting Information S1). However, it is too noisy to identify them through the radio signals and beyond the scope of this study to further investigate them.

Despite one special case far from the other multiple‐pulse BLUEs, all the single‐pulse and multiple‐pulse BLUEs are well separated into two clusters. The primary BLUEs of the multiple‐pulse BLUEs in our study have relatively weaker current moments and amplitudes than those corresponding to the single‐pulse BLUEs. Note that the statistical significance of the distribution is discussed at the footnote of Figure S30 in Supporting Information S1.

This is reminiscent of initiation‐type NBEs, which also have weaker source currents. We emphasize however that all the multiple‐pulse NBEs that we analyzed are isolated NBEs, separated from any IC or CG lightning discharges detected by either GLD360 or the 777.4‐nm band of MMIA within at least 100 ms. They are also located at relatively high altitudes nearby cloud tops, unlike the INBEs normally located deeply inside the thundercloud (Smith et al., 2004; Wu et al., 2014).

## 4 Discussion and Conclusions

Our results suggest that a fraction of so‐called isolated NBEs, which do not initiate leader activity and are therefore not the starting event of a lightning flash, nevertheless trigger subsequent breakdown activity. We now discuss some implications of these findings.

Turning first to the thunderstorm environment that surrounds the analyzed multiple‐pulse BLUEs, we notice from the lightning distribution in Figure 1 that the rate of negative CGs exceeds that of positive ones by about a factor three, which suggests that the thunderstorm has a dipole‐like electrical structure with the positive charge above the negative charge (Wilson, 1956). However, the charge structures can be more complex in the convective region of the thunderstorm (Stolzenburg et al., 1998). Both IC and CG flash rates vary dramatically during the time interval when the BLUEs occurred.

The negative CG rate decreases sharply as the rate of the positive ICs increases, and later the rates of all ICs and CGs start to increase. The dramatic change of the lightning rates suggests that the lightning discharges are produced inside a thunderstorm with deep convective updrafts (Petersen & Rutledge, 1998; Wiens et al., 2005). The ring structures shown in Figures S6 and S8 in Supporting Information S1 further illustrate that there is a cloud turret extending above the cloud top surface during the occurrence interval of the BLUEs (Luque et al., 2020). One hypothesis for this is that the positive NBEs are produced between the positive charge lifted to relatively high altitude by the strong updraft and the negative screening charge layer which lies close to the overshooting region of the cloud (MacGorman et al., 2017).

Interestingly, the primary BLUEs of the multiple‐pulse BLUEs in our study have relatively weaker current moments and amplitudes than those corresponding to the single‐pulse BLUEs. However, our study was limited to a single thunderstorm; additional studies are required to determine whether this conclusion can be generalized.

Our results are connected to the problem of lightning initiation. If our interpretation is correct, there is an intermediate class of electrical discharges between those that are fully isolated and those that initiate a leader. This would be the class of breakdowns that trigger subsequent electrical discharges which do not promote to leaders. It is unclear whether these discharges, with a primarily horizontal orientation and associated with weak radio pulses, are similar to the primarily vertical fast breakdown events described previously in the literature (Huang et al., 2021; N. Y. Liu & Dwyer, 2020; Lyu et al., 2019; Rison et al., 2016; Tilles et al., 2019). It is also unknown whether NBE‐initiated leaders are initiated by horizontal breakdowns. These questions should be addressed by future research.

## Supporting information

Supporting Information S1

## Data Availability

The Modular Multispectral Imaging Array (MMIA) level 1 data is proprietary and not currently available for public release. Interested parties should direct their request to the ASIM Facility Science Team (FST). ASIM data request can be submitted through: https://asdc.space.dtu.dk by sending a message to the electronic address asdc@space.dtu.dk. The Himawari‐8 gridded data in this study is public to the registered users and supplied by the P‐Tree System, Japan Aerospace Exploration Agency (JAXA)/Earth Observation Research Center (EORC) (https://www.eorc.jaxa.jp/ptree/). The data that support the findings of this study are openly available in https://doi.org/10.5281/zenodo.6123813. The Cloudscat Monte Carlo simulation code (Luque et al., 2020) is available at https://github.com/aluque/CloudScat.jl and https://doi.org/10.5281/zenodo.3842787. The stanford Full‐Wave Method (StanfordFWM) code (Lehtinen & Inan, 2008, 2009) is available at https://gitlab.com/nleht/stanfordfwm/.
