# Supplementary material for: Multi‐Pulse Corona Discharges in Thunderclouds Observed in Optical and Radio Bands
Source: Geophys Res Lett. 2022 Jul 8;49(13):e2022GL098938. doi: 10.1029/2022GL098938 (PMC9539966; doi:10.1029/2022GL098938)
Supplement: Supplementary file 1 — Supporting Information S1 [file GRL-49-e2022GL098938-s001.pdf]

Supplemental Material for "Multi-pulse corona discharges in thunderclouds observed in optical and radio bands"

Dongshuai Li[1], Alejandro Luque[1], Nikolai G. Lehtinen[2], F. J. Gordillo-Vázquez[1], Torsten Neubert[3], Gaopeng Lu[4], Olivier Chanrion[3], Hongbo Zhang[5], Nikolai Østgaard[2], Víctor Reglero[6]

[1]Instituto de Astrofísica de Andalucía (IAA), CSIC, Granada, Spain.

[2]Birkeland Centre for Space Science, Department of Physics and Technology, University of Bergen, Bergen, Norway.

[3]National Space Institute, Technical University of Denmark (DTU Space), Kongens Lyngby, Denmark.

[4]CAS Key Laboratory of Geospace Environment, University of Science and Technology of China, Hefei, China.

[5]Key Laboratory of Middle Atmosphere and Global Environment Observation (LAGEO),

Institute of Atmospheric Science, Chinese Academy of Sciences, Beijing, China.

[6]Image Processing Laboratory, University of Valencia, Valencia, Spain.

Contents of this file

1. Text S1

2. Figures S1 - S30

Text S1: Methodology

MMIA observation simulation

In the simulation, we assumed the optical BLUE sources are impulsive and localized point sources inside a homogeneous isotropic cloud. The scattering and absorption processes of the photons propagating through the cloud are evaluated based on two different approaches including an analytical diffusion model and a Monte Carlo simulation (Luque et al., 2020).

We first fit the 337-nm photometer signal of MMIA to infer the depth (relative to the cloud top) L of point-like optical sources inside a homogeneous and infinite cloud. To simplify the simulation, we neglect Rayleigh scattering and background absorption by adopting a homogeneous collision rate $\nu = cN_d Q_{ext} \pi R^2$, where N_d is a droplet number density with particle radius R.

Soler et al. (2020) proposed a simplified analytical expression based on the diffusion approximation proposed by Koshak et al. (1994), named first-hitting-time model, to infer the depth (relative to the cloud top) of the point-like optical sources located deep inside the cloud. However, here we add more details by using equation (27) of Luque et al. (2020) to include Mie scattering. Mie scattering corresponding to the wavelength of 337 nm is characterized by three parameters: the scattering asymmetry parameter $g = 0.88$, the respective extinction coefficient $Q_{ext} = 2.06$ and the single-scattering albedo $\omega_0 = 0.99$ which is close to unity and describes the probability that a photon re-emits after a scattering event. These parameters are obtained by solving the Mie problem with the open source MieScatter.jl code (Wilkman, 2013; Li et al., 2020) .

The total flux per unit time of single-pulse BLUEs $F_{\text{single}}(t)$ for the analytical diffusion model is (see more details in (Luque et al., 2020)):

$$F_{\text{single}}(t) = \frac{e^{-t/\tau_A - \tau_D/t}}{\sqrt{\pi}\tau_D}(t/\tau_D)^{-3/2} \tag{1}$$

where the photon absorption time $\tau_A = \frac{1}{\nu(1-\omega_0)}$ and the characteristic time $\tau_D(t) = \frac{L^2}{4D}$ with the depth L and the diffusion coefficient $D = \frac{c^2}{\nu(1-\omega_0)}$.

The equation for multiple-pulse BLUEs, denoted as $F_{\text{multiple}}(t)$, is a sum of $F_{\text{single}}(t)$ as follows:

$$F_{\text{multiple}}(t) = \sum_{i=1}^{M} F_{\text{single}}(t) \tag{2}$$

where M is the number of the BLUE pulses used in the fitting process.

We further simulate the 337-nm photometer signal and the corresponding camera image detected by MMIA using a Monte Carlo code CloudScat.jl (Luque et al., 2020) by considering a localized optical point source inside a homogeneous cloud at an altitude that spans from 7 km to the cloud top boundary. The depth L of the optical source is derived from the analytical diffusion model with the scattering parameters listed in Table 2.

FWM simulation

We simulate the radio waveform of both NBEs and the subsequent pulse trains of the multiple-pulse BLUEs using the Stanford Full Wave Method (StanfordFWM) code of Lehtinen & Inan (2008, 2009). The source is assumed to be a vertical dipole for the NBEs and a horizontal dipole for the subesequent pluse trains with the current moment of 1 A m located at an altitude of 10 km (selected for simplicity, neglecting the small differences with the estimated source altitudes) emitting at frequencies between 10 kHz to 100 kHz. The waveform of the current moment is assumed to be the bi-Gaussian function:

$$I(t) = I_0(e^{-t^2/\tau_1{}^2} - e^{-t^2/\tau_2{}^2}), \tag{3}$$

where the rise time τ_1 and the fall time τ_2. The electric current moment for the NBEs is $I_0 = [0, 0, 1]$ corresponding to a vertical dipole along z-axis. The electric current moment for subsequent pulse trains is $I_0 = [\sqrt{2}/2, \sqrt{2}/2, 0]$, i.e., represents a horizontal dipole with an angle of 45° with respect to the positive x-axis. Note that the angle of 45° here only represent an example of the cases since we don't know the exact angle of the horizontally oriented sources. The ionosphere is assumed to be horizontally stratified at altitudes between 0 km to 100 km and treated as a magnetized plasma. In order to reflect the real propagation geometry, we assumed the propagation in positive x-direction corresponding to the north component of the geomagnetic field. According to the International Geomagnetic Reference Field (IGRF) model (Alken et al., 2021), the geomagnetic field in our case has the total intensity about 42 000 nT along the positive y-axis. In the simulation, we only consider the electrons since the effect of the ions can be neglected in the lower ionosphere in the frequency range of interest. The electron density profile is obtained using the International Reference Ionosphere (IRI) model (Bilitza et al., 2014) at the location of the BLUEs at 17:50:00 UTC on April 30, 2020. The ground is assumed to be perfectly conducting without considering the effect of the ground conductivity due to the BLUEs occurred over the ocean. By following the geometry of the observation, the observed sensor at Malaysia is located at about 500 km away from the BLUEs (see figure 1(b)). After the results of the FWM modeling at different frequencies are obtained, we applied the inverse Fourier transform to calculate the time-domain waveforms of the x and y components of the

magnetic field at the observation point. The comparison between the StanfordFWM results and the observation are shown in figure 3.

VLF/LF sferic simulation

We estimate the current moment $M_i(t)$ for the primary BLUE pulse of the multiple-pulse BLUEs based on the azimuthal magnetic field component B_ϕ measured by the ground-based very low frequency/low frequency (VLF/LF) sensor nearby Malaysia. In the calculation, we assumed the source as a vertical dipole located at an altitude of H away from the sensor at a distance of d (see Table 2). The ground is assumed to be perfectly conducting because the VLF waves propagation from the BLUEs occurred over the ocean. We calculate the azimuthal magnetic field component B_ϕ by using Uman's equation (Uman et al., 1975) and compare it with the ground wave signals measured by the VLF/LF sensor nearby Malaysia. The source current moments can be inferred by solving the inverse convolution problem (Cummer & Inan, 2000; Cummer, 2003):

$$B(t) = \int_{-\infty}^{\infty} M_i(t)h(t-\tau)d(\tau), \tag{4}$$

where B is the measured magnetic field waveform, M_i is the source current moment and $h(t)$ is the propagation response evaluated from the modeling results of Uman's equation. In the modeling, the waveform of the source current is also assumed to be the bi-Gaussian function in equation (3). Figure S28 shows the comparison between the simulation and observation for all the multiple-pulse BLUE cases. The inferred current moments M_i and the cumulative charge moments M_q of all multiple-pulse BLUEs are presented in Figure S29.

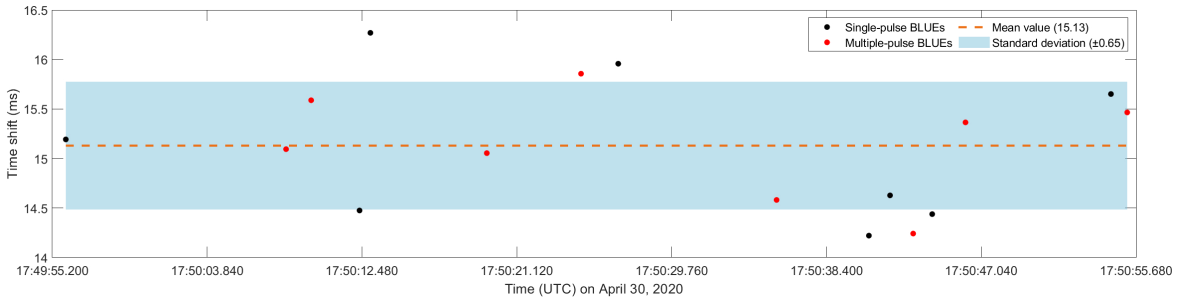

Figure S1. The systematic time shift of MMIA with respect to the ground-based VLF/LF radio signals calculated by using 16 BLUEs (8 single-pulse BLUEs (black dots) and 8 multiple-pulse BLUEs analyzed in the paper (red dots)) simultaneously detected by the 337-nm photometer and its filtered camera of MMIA and the ground-based VLF/LF sensor nearby Malaysia. The mean value of the MMIA time shift is about -15 ms with the standard deviation ±0.65 ms.

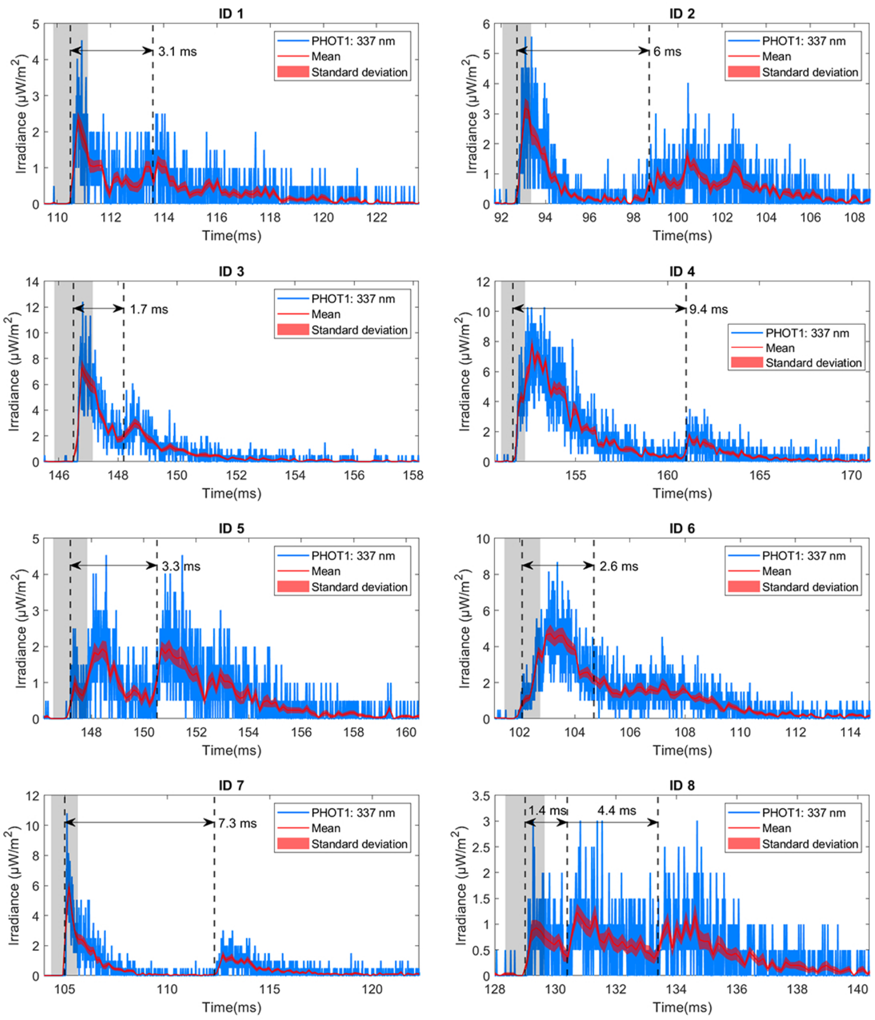

Figure S2. The moving average of 15 data points (corresponding to 150 μs) of the 337-nm photometer signal. The mean and standard deviation of the sample mean are marked in the red solid line and its shaded band. The start time (refer to source) for NBE and its subsequent pulse is marked in dashed black line with ±0.65 ms uncertainty (gray shadowed region). The event with ID 6, where the two pulses overlap but are identifiable nevertheless, is corresponding to one special case where the subsequent pulse trains look very much like a negative NBEs, however, it is too noisy to identify it through the radio signals (see Fig.S24).

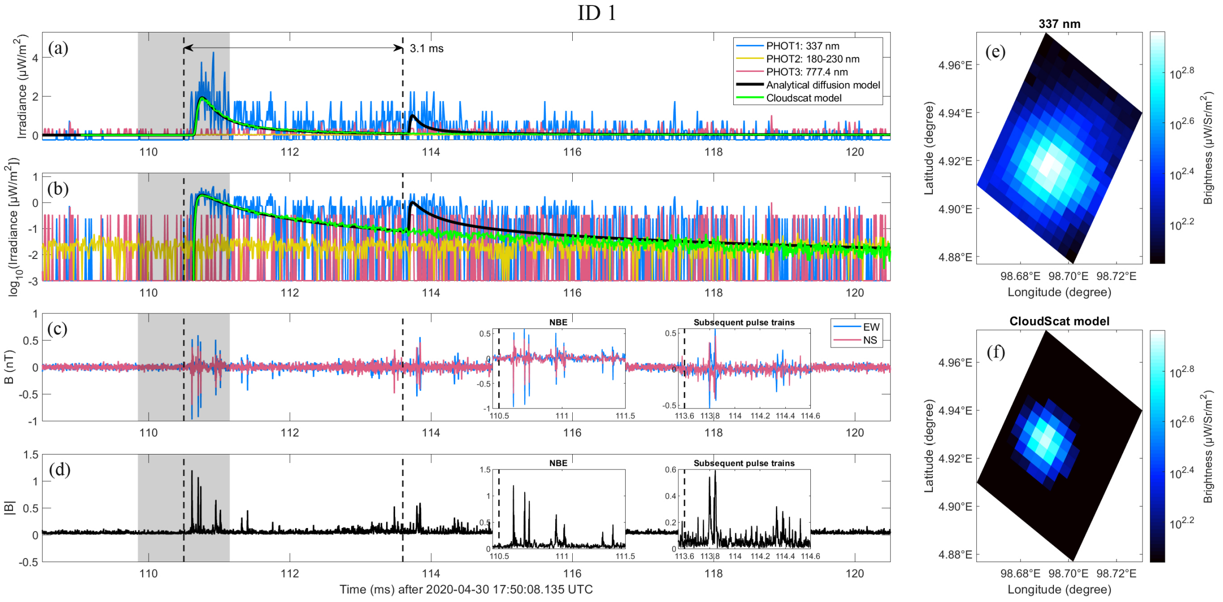

Figure S3. Comparison between MMIA photometer irradiance (blue: 337 nm, yellow: 180-230 nm and red: 777.4 nm) and the modeling results of the analytical diffusion model (black) and Cloudscat model (green) on a linear (a) and logarithmic (b) scale along with the North-south and East-west magnetic field components B_{NS} and B_{EW} (c), and its norm $|B| = \sqrt{B_{\mathbf{NS}}^2 + B_{\mathbf{EW}}^2}$ (d) from the ground-based VLF/LF sensor nearby Malaysia for event 1. The image detected by 337-nm filtered camera of MMIA (e) and the simulated image of Cloudscat model (f). The start time (refer to source) for NBE and its subsequent pulse is marked in dashed black line within the time difference 3.1 ms with ±0.65 ms uncertainty (gray shadowed region). The inset zoom figures for both primary NBE and its subsequent pulse trains are also given in the figure.

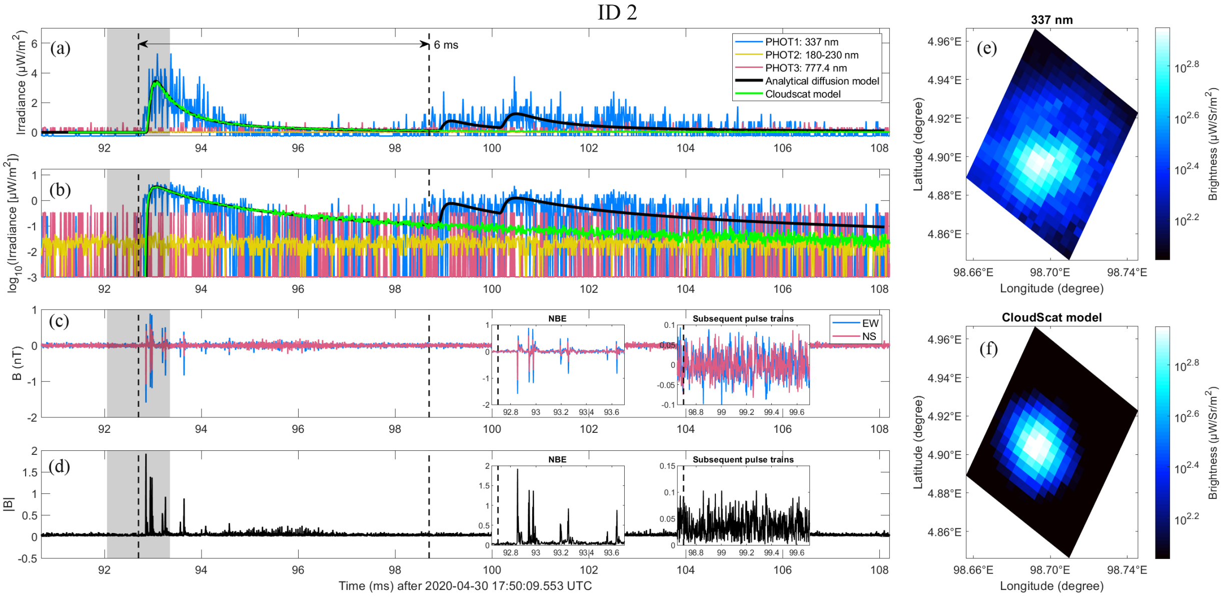

Figure S4. Similar to Figure S3, but for event 2.

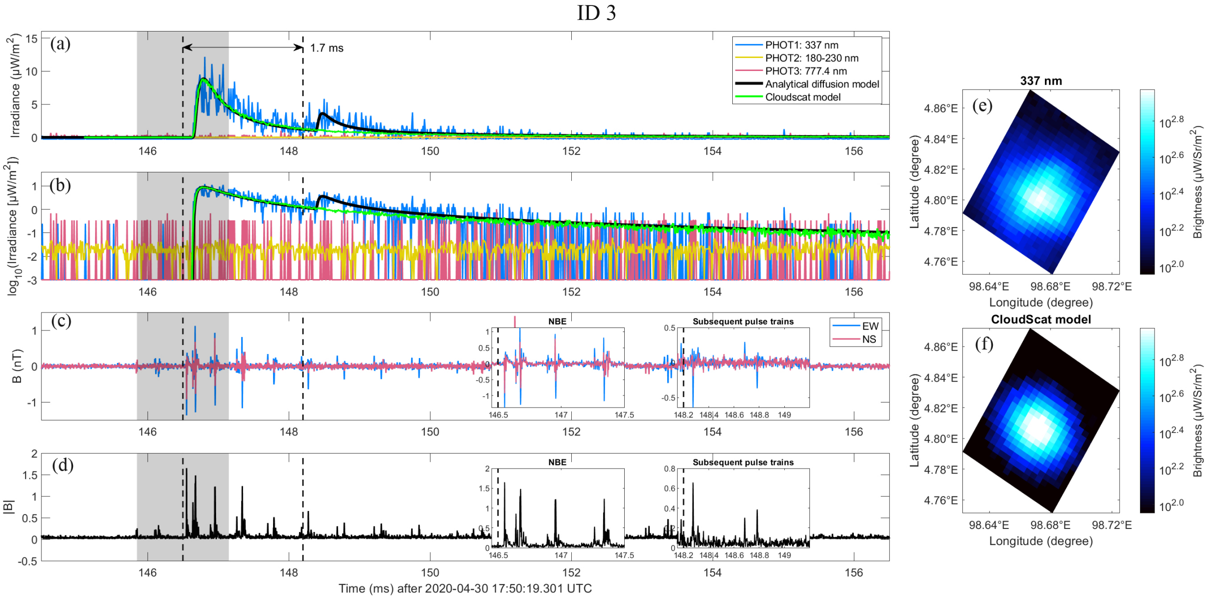

Figure S5. Similar to Figure S3, but for event 3. For this case, the subsequent pulse trains are not obvious and might overlap with the multiple-hop ionospheric reflections of NBEs due to the complex condition of the magnetized plasma in the lower ionosphere.

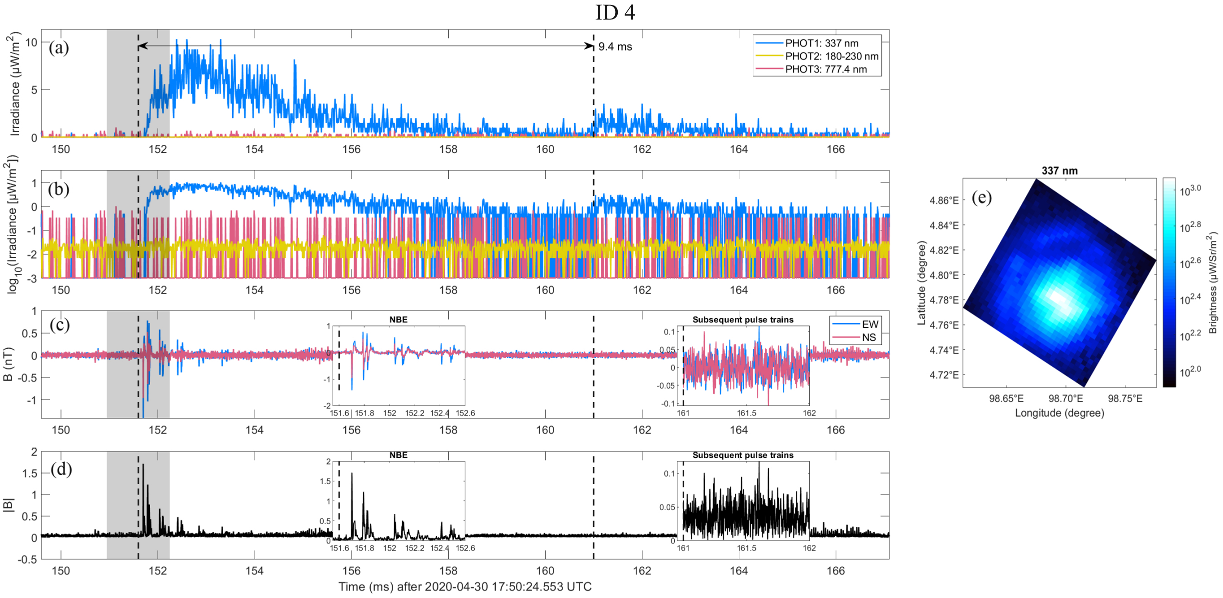

Figure S6. Photometer irradiance (blue: 337 nm, yellow: 180-230 nm and red: 777.4 nm) of MMIA on a linear (a) and logarithmic (b) scale along with the magnetic field components B_{NS}, B_{EW} (c), and its norm $|B| = \sqrt{B^2_{\mathbf{NS}} + B^2_{\mathbf{EW}}}$ (d) detected from the ground-based VLF/LF sensor nearby Malaysia for event 4. The image detected by 337-nm filtered cameras of MMIA (e). The start time (refer to source) for NBE and its subsequent pulse is marked in dashed black line within the time difference 9.4 ms with ±0.65 ms uncertainty (gray shadowed region). The inset zoom figures for both primary NBE and its subsequent pulse trains are also given in the figure.

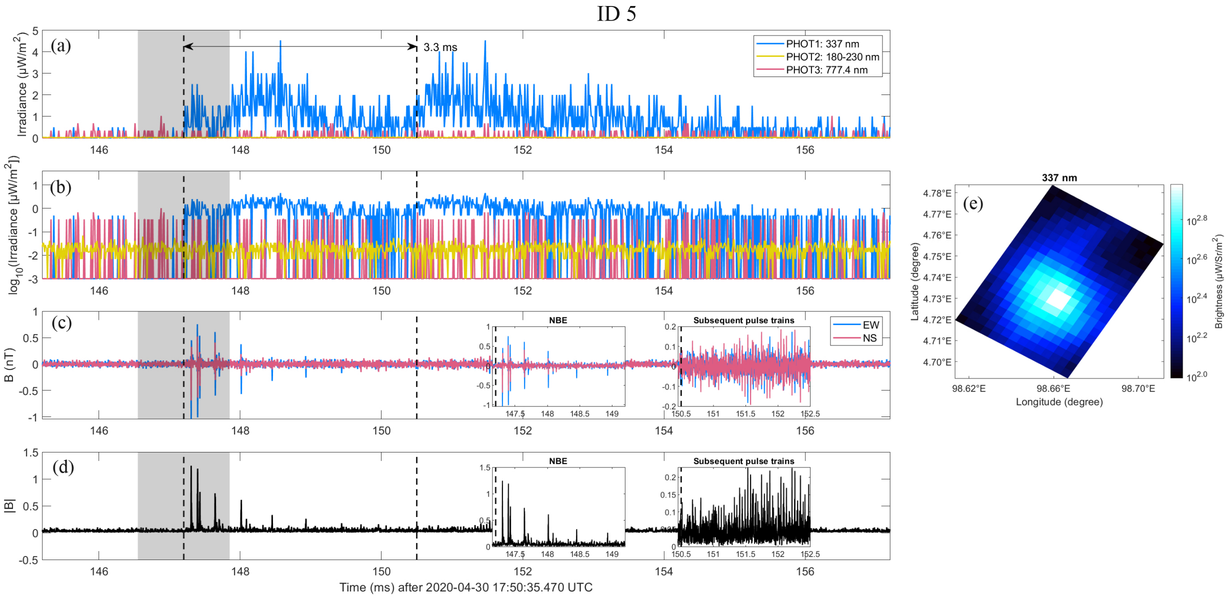

Figure S7. Similar to Figure S6, but for event 5.

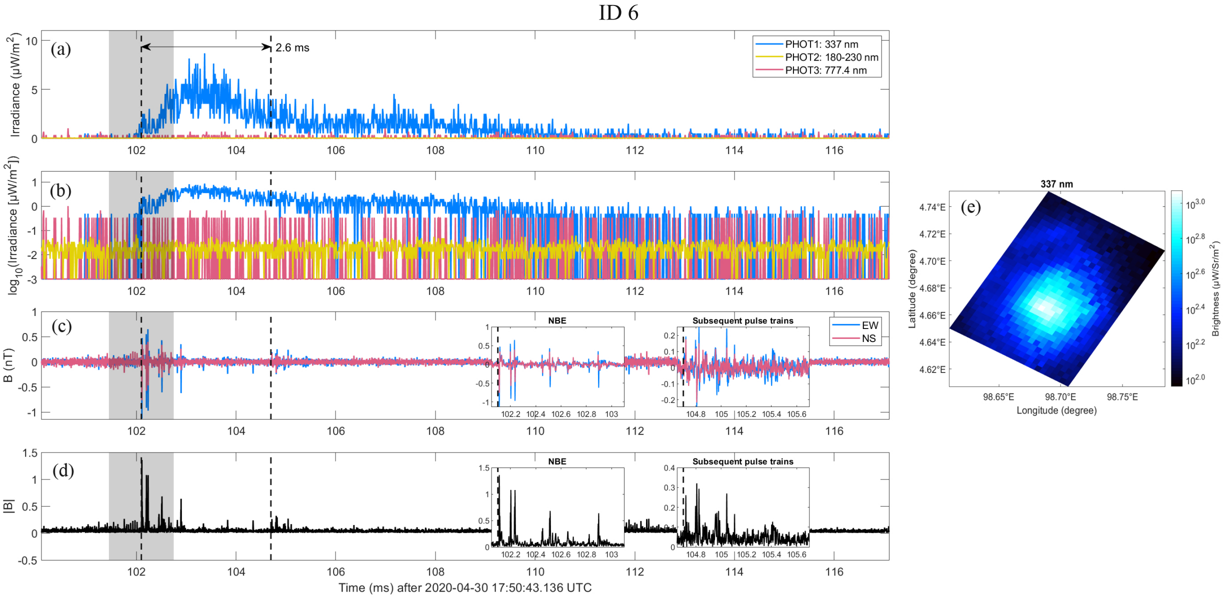

Figure S8. Similar to Figure S6, but for event 6.

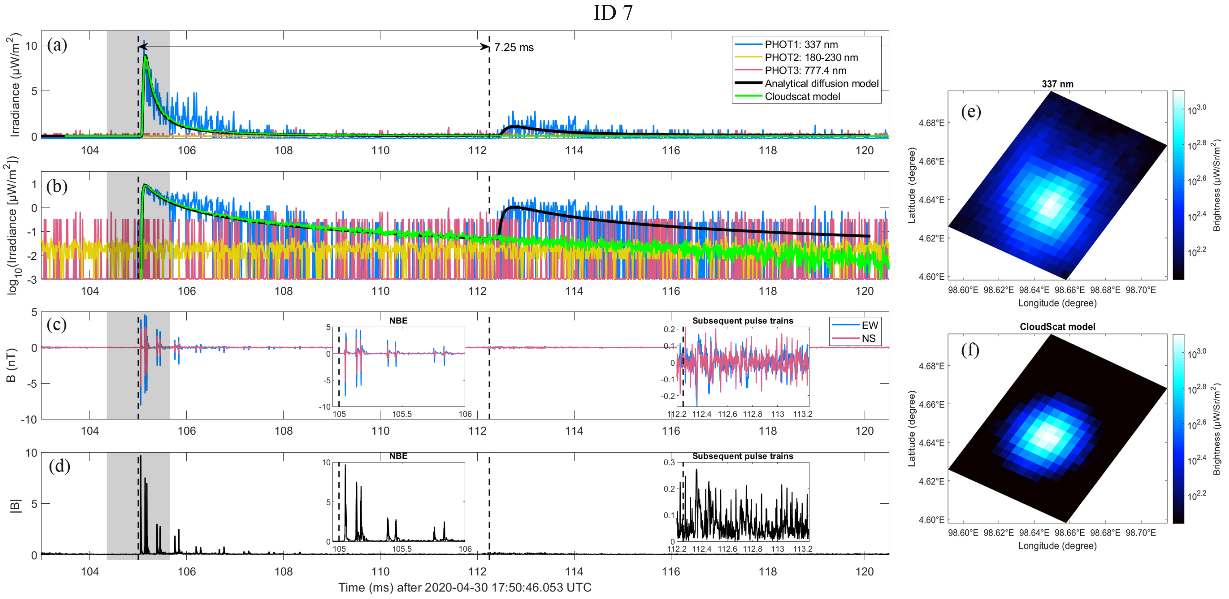

Figure S9. Similar to Figure S3, but for event 7.

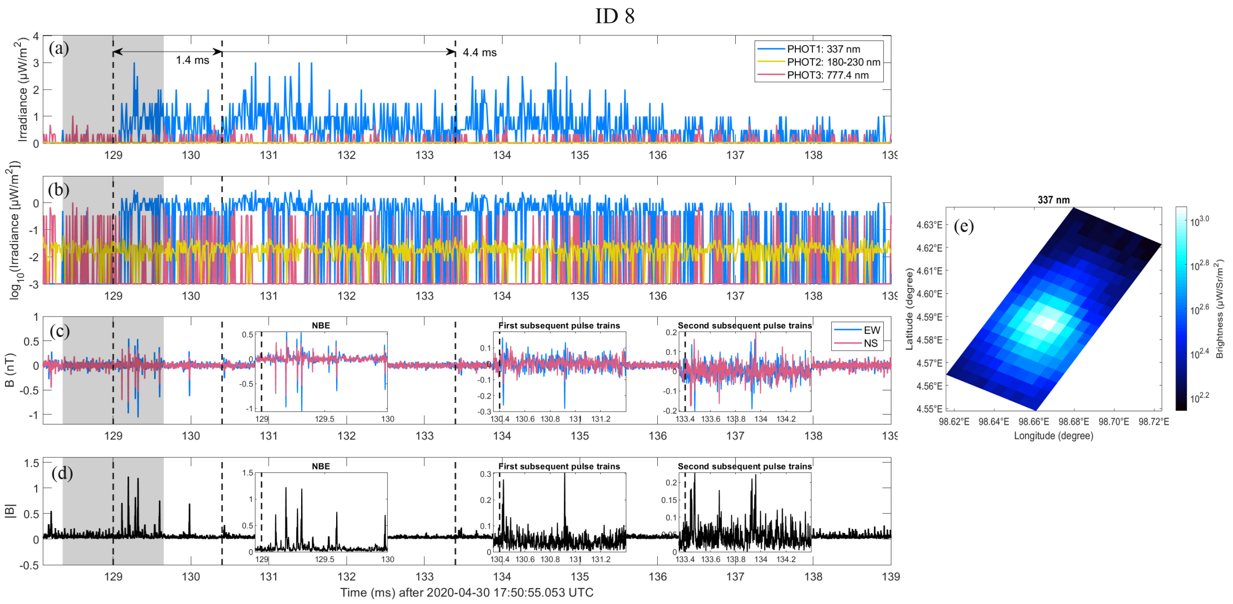

Figure S10. Similar to Figure S6, but for event 8. Note that the zoom-in of the first and second subsequent pulses trains within time difference 1.4 ms and 4.4 ms are also shown in the figure. For this case, the first subsequent pulse trains are not obvious and might overlap with the multiple-hop ionospheric reflections of NBEs due to the complex condition of the magnetized plasma in the lower ionosphere.

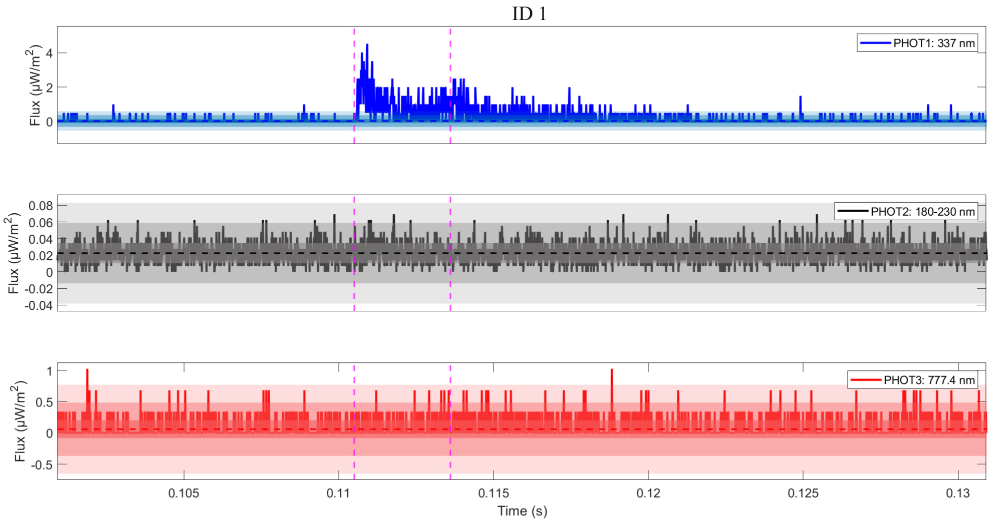

Figure S11. The statistical significance of the photometer signals for event 1 (Blue: 337-nm, Black: 180-230 nm and Red: 777.4 nm). The pink vertical dashed line marked the primary and secondary BLUE pulses. The horizontal dashed line is empirical average μ of the background noises with the shaded bands indicating $\mu \pm \sigma$, $\mu \pm 3\sigma$ and $\mu \pm 5\sigma$. The empirical average μ and standard deviation σ for the background signal are calculated by using 1000 data points (10 ms) before the first primary BLUE begins.

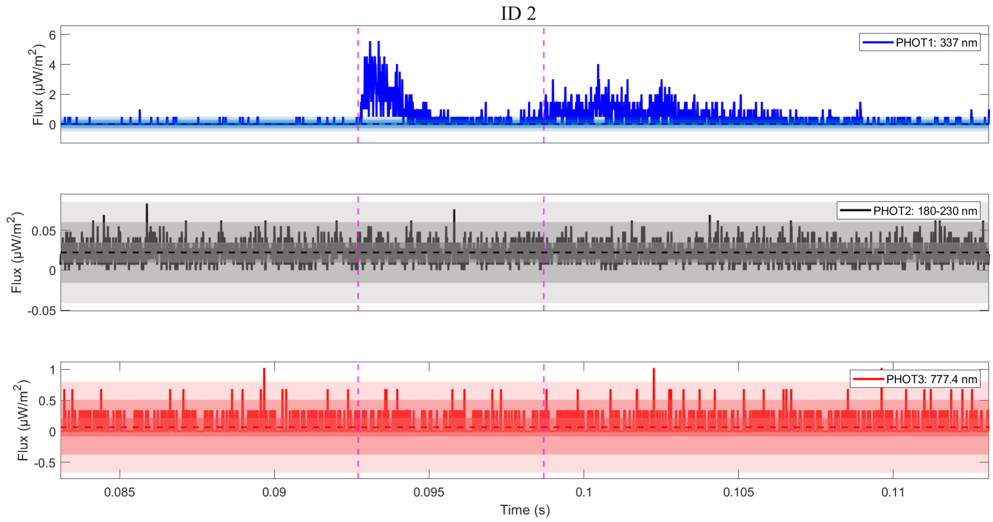

Figure S12. Similar to Figure S11, but for event 2.

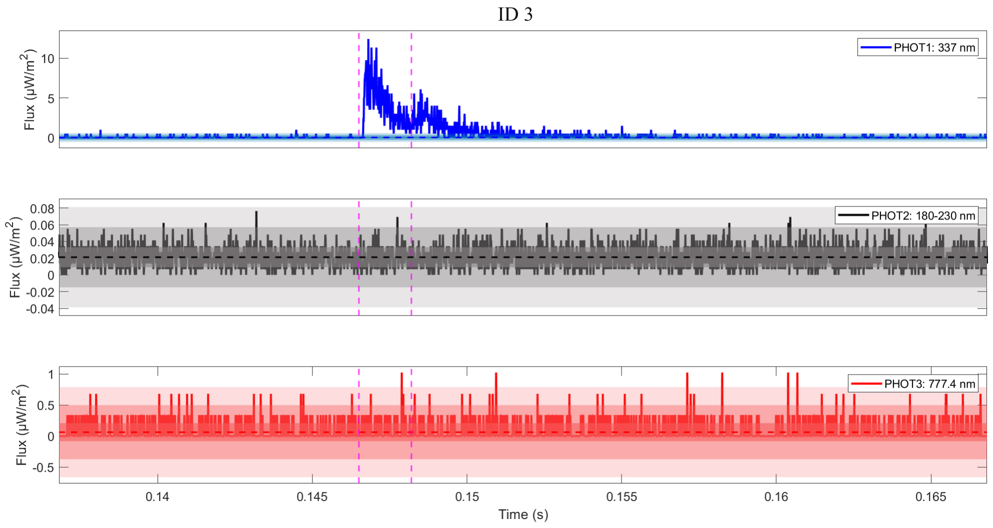

Figure S13. Similar to Figure S11, but for event 3.

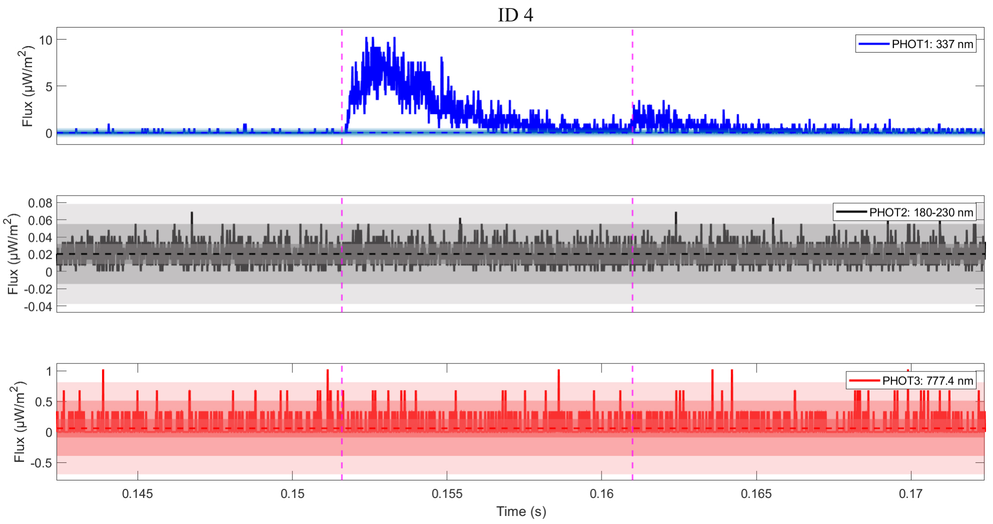

Figure S14. Similar to Figure S11, but for event 4.

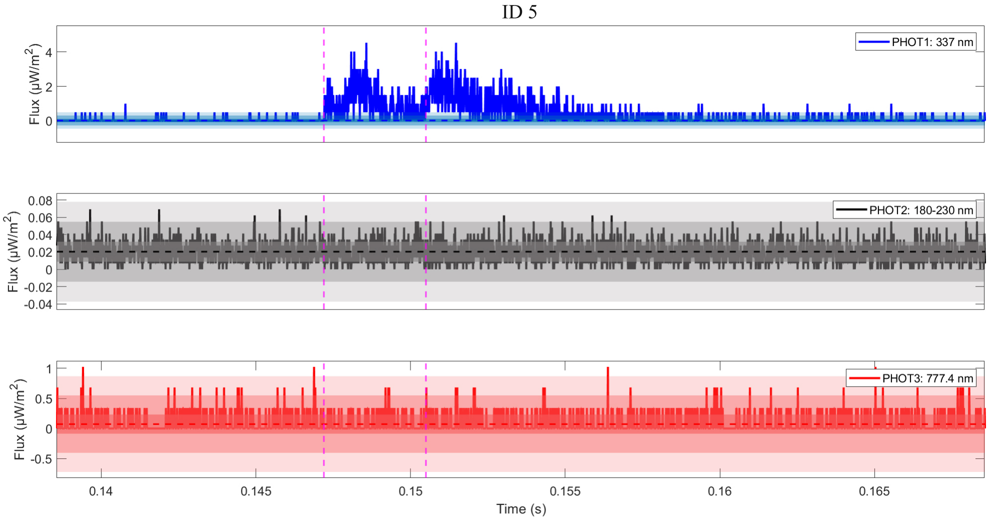

Figure S15. Similar to Figure S11, but for event 5.

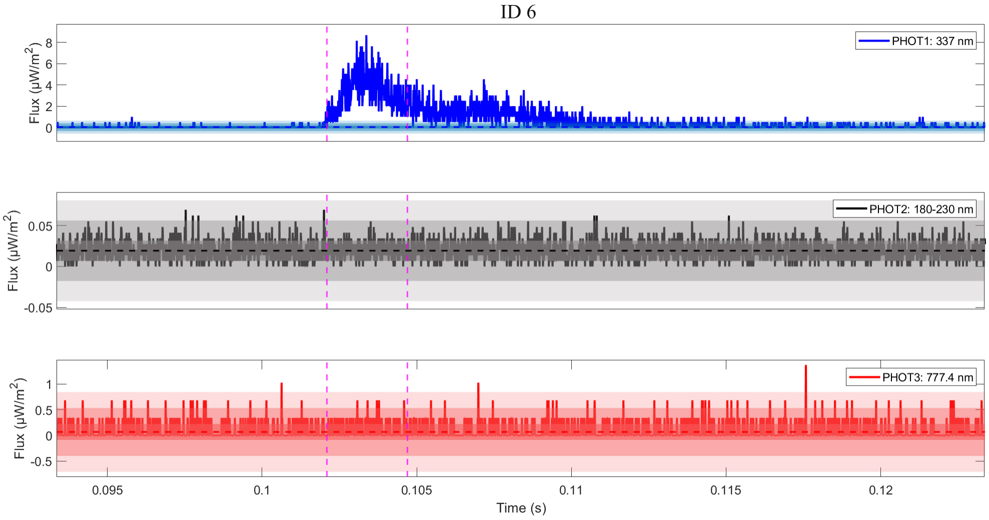

Figure S16. Similar to Figure S11, but for event 6.

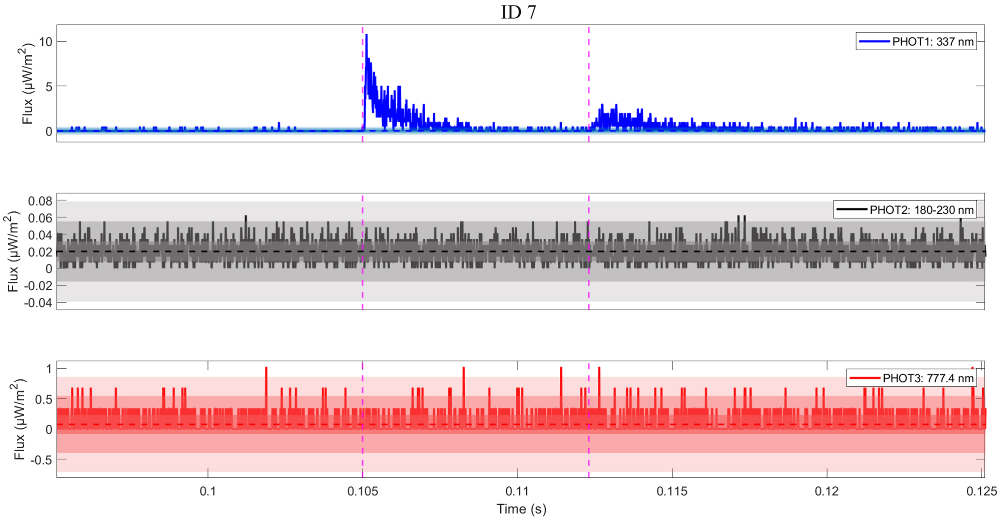

Figure S17. Similar to Figure S11, but for event 7.

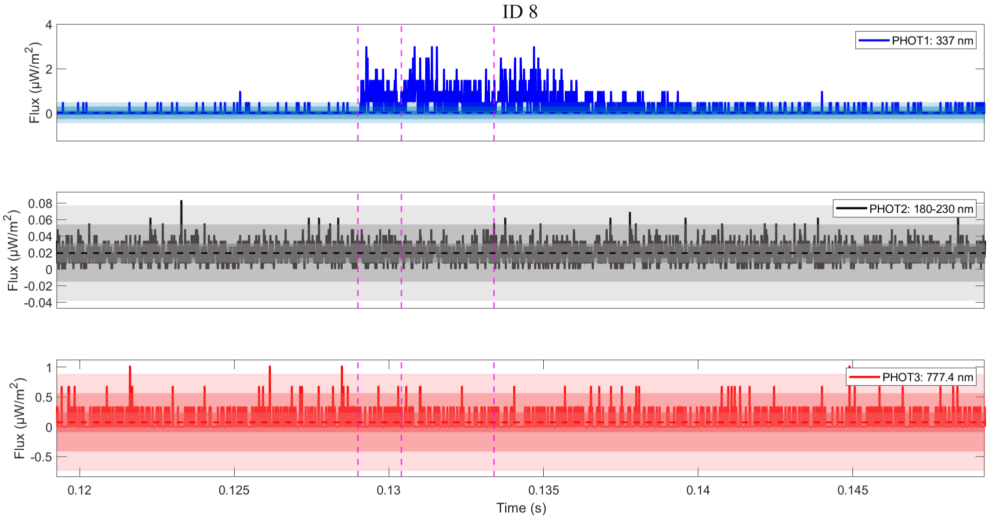

Figure S18. Similar to Figure S11, but for event 8.

ID 1

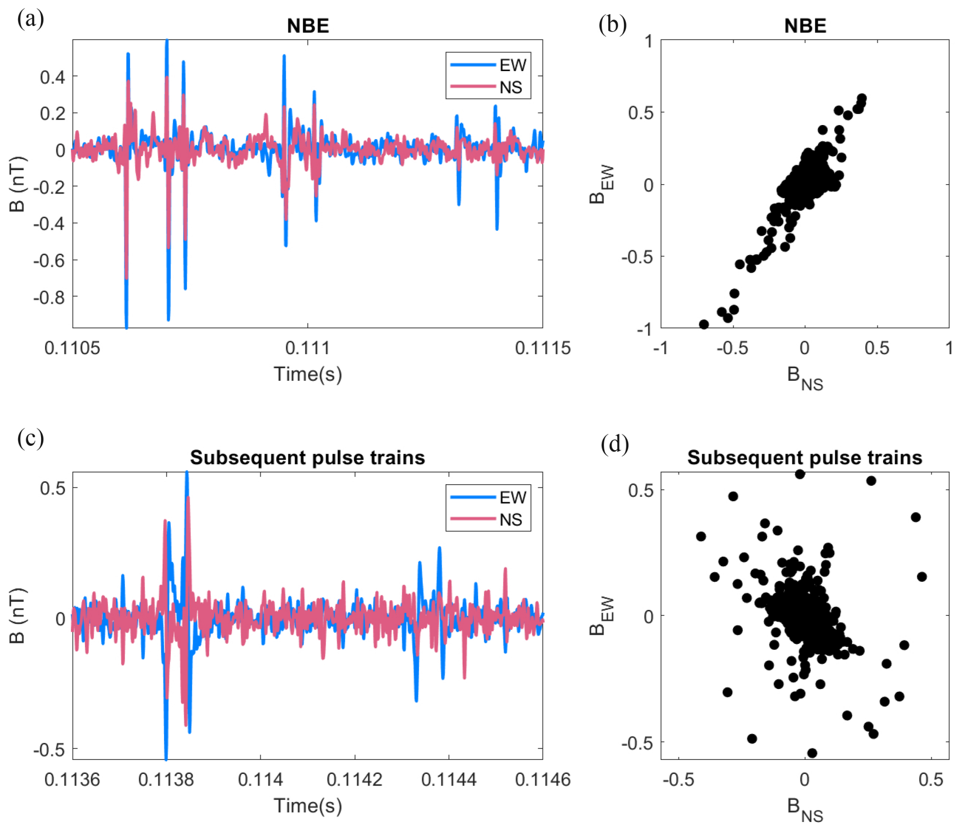

Figure S19. The waveform of the North-south and East-west magnetic field component (B_{NS} and B_{EW}) and the correlation between them for both NBE (a,b) and its subsequent pulse trains (c,d) of the multiple-pulse BLUE for event 1.

ID 2

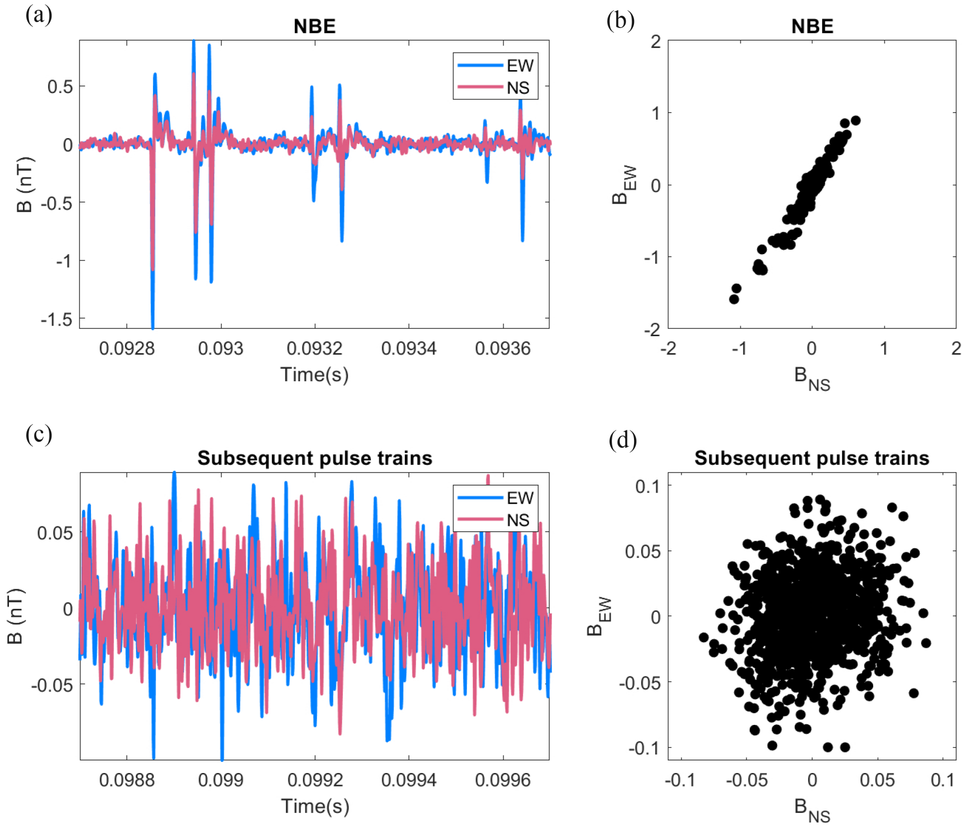

Figure S20. Similar to Figure S19, but for event 2.

ID 3

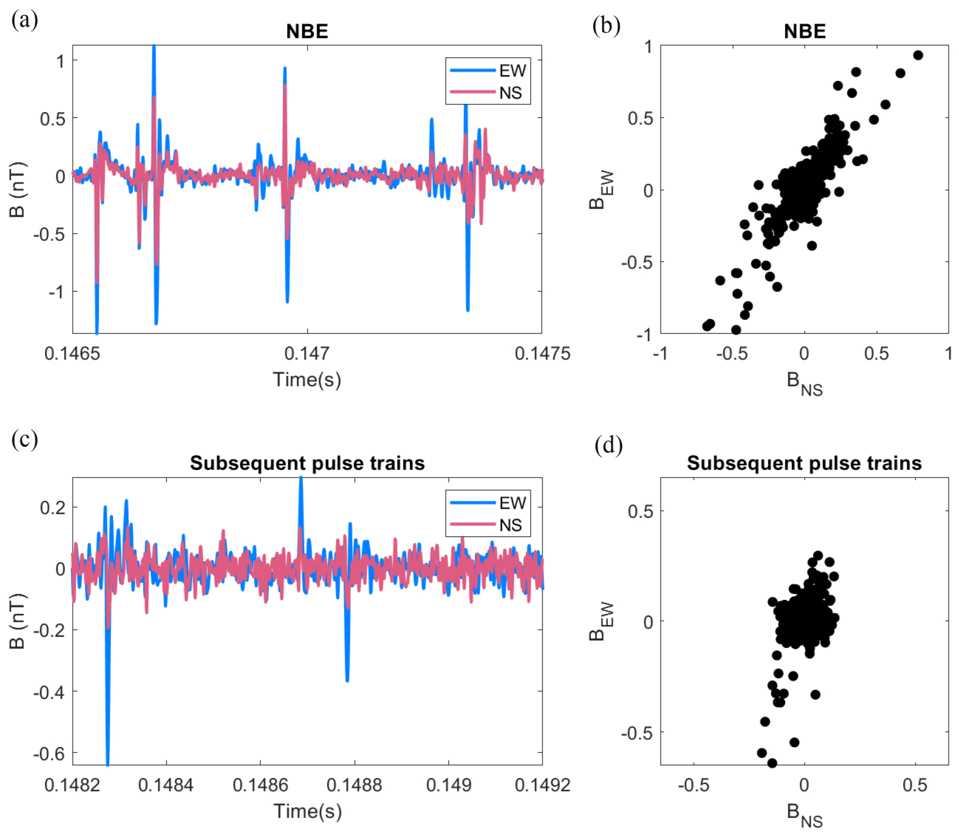

Figure S21. Similar to Figure S19, but for event 3.

ID 4

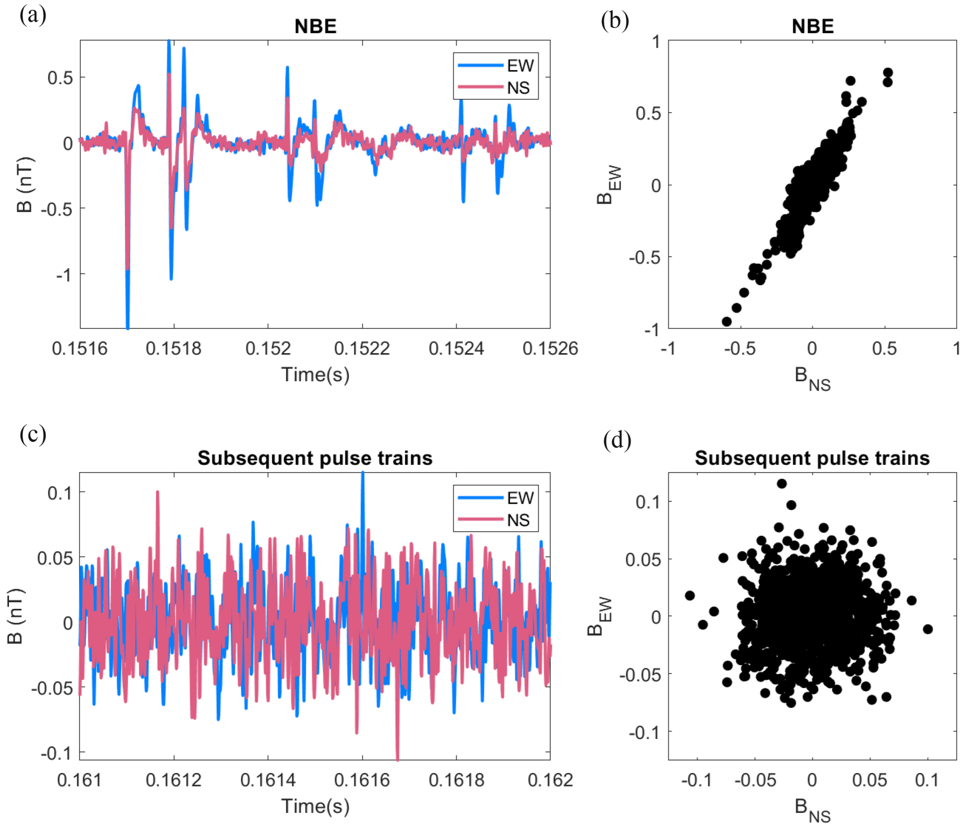

Figure S22. Similar to Figure S19, but for event 4.

ID 5

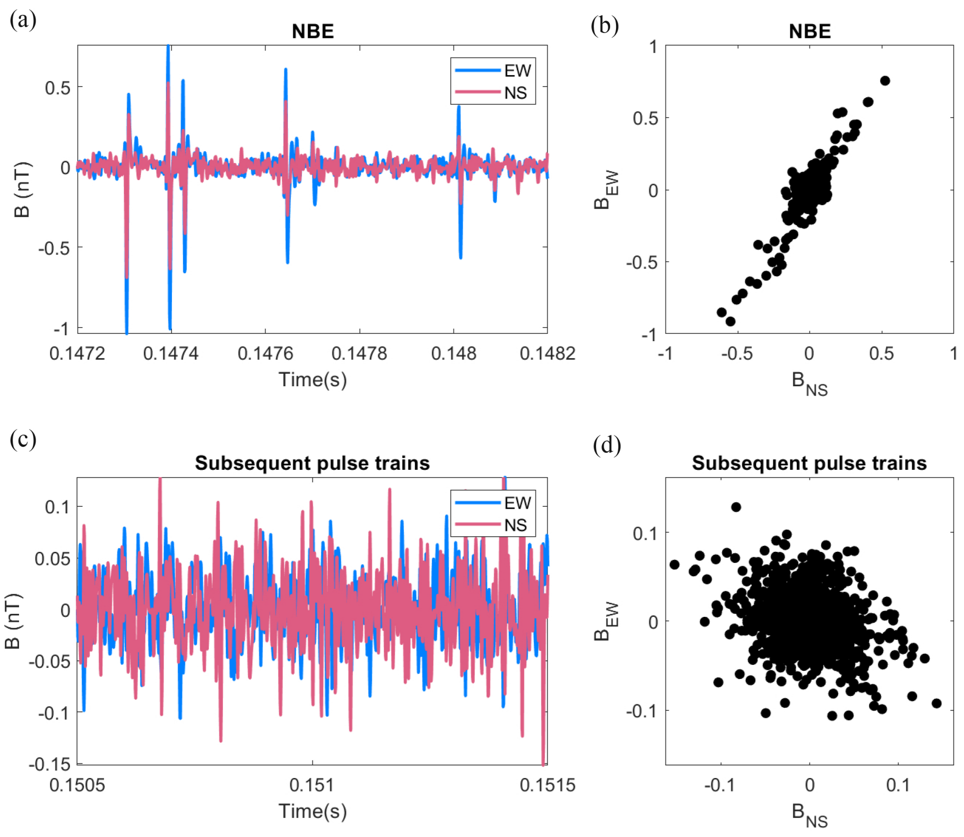

Figure S23. Similar to Figure S19, but for event 5.

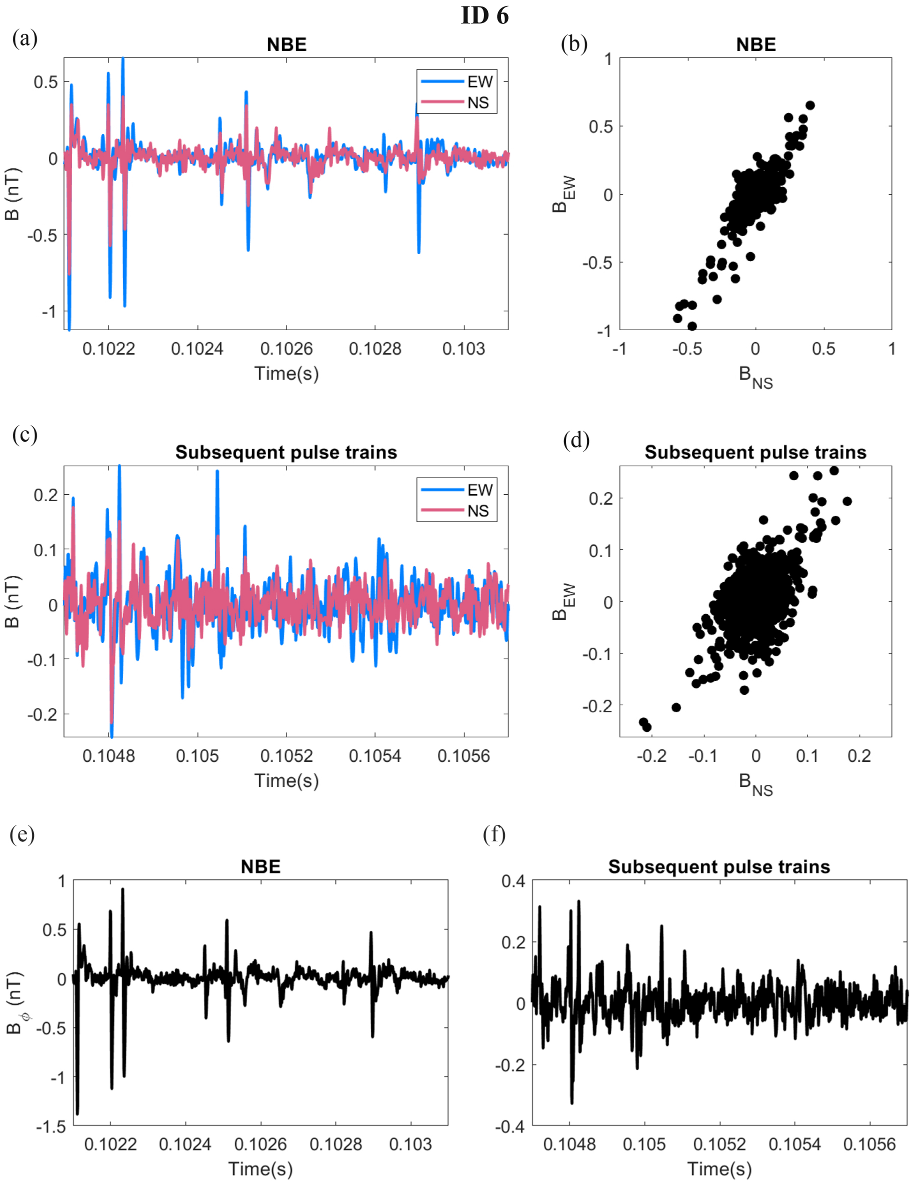

Figure S24. Similar to Figure S19, but for event 6. The radio signals for the Azimuthal magnetic fields for both NBE and its subsequent pulse trains are further given in (e) and (f). Note that, as shown in (d), the subsequent pulses trains show a similar linear-like pattern comparing with NBE pulses. It is due to the subsequent pulse trains for the event 6 seems like a negative NBE, however, it is too noisy to identify it through the radio signals.

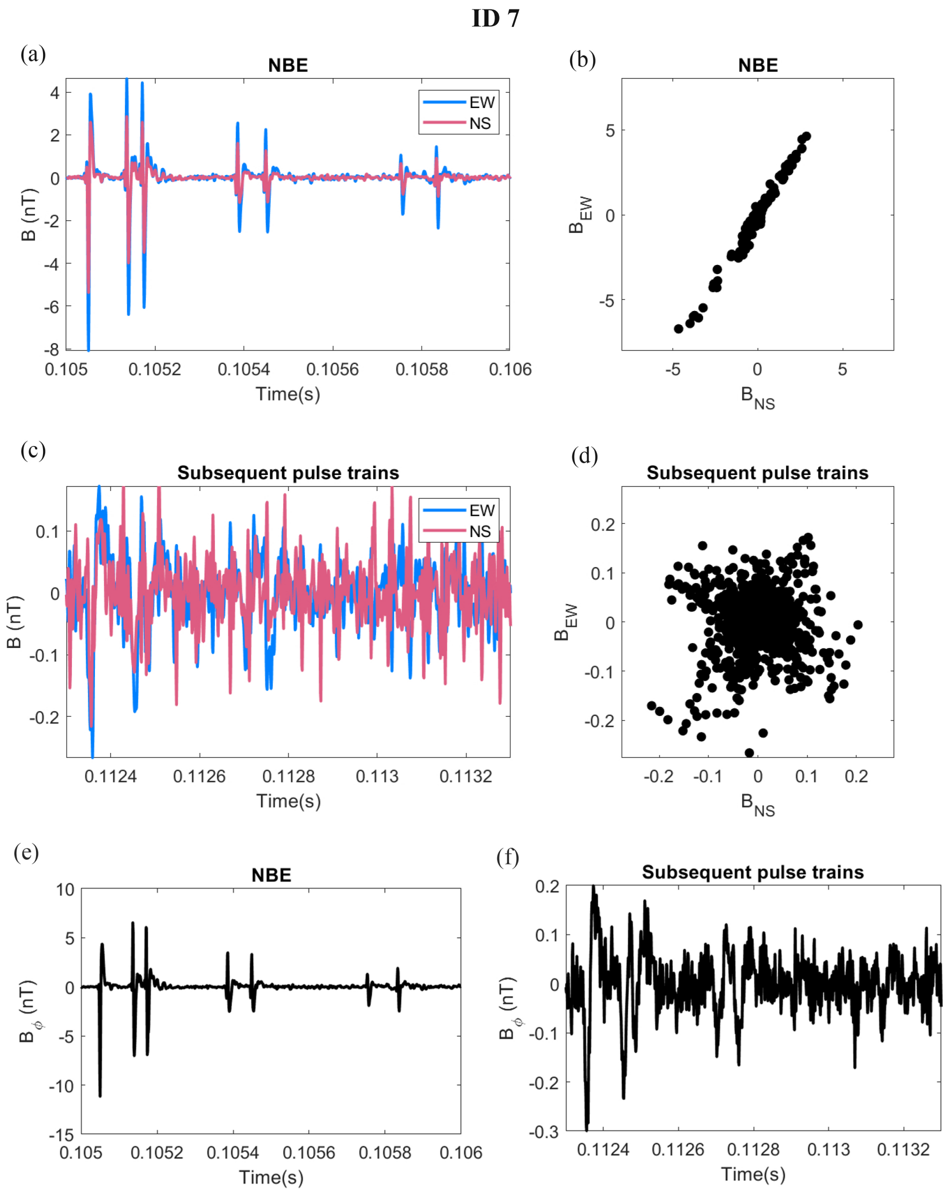

Figure S25. Similar to Figure S19, but for event 7. For this special case, the radio signals for the Azimuthal magnetic fields for both NBE and its subsequent pulse trains are further given in (e) and (f). The subsequent pulse trains for event 7 seems to be "NBE-like" events, which might two NBE events occurred closely in time, however, it is too noisy to identify it through the radio signals.

ID 8

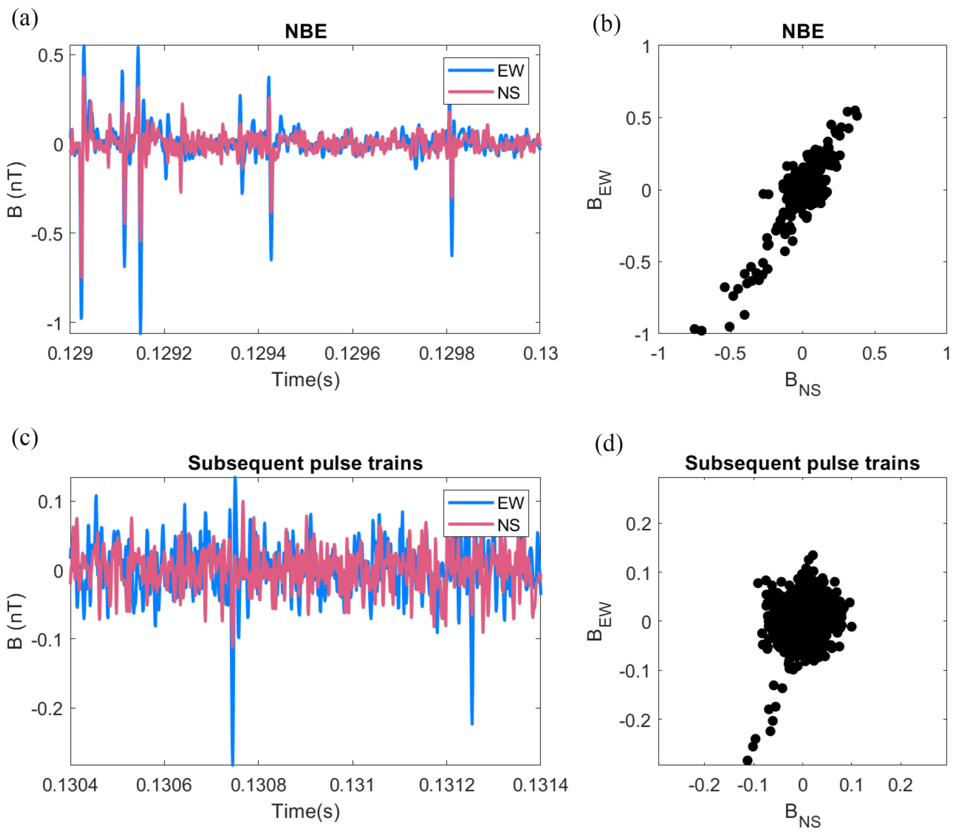

Figure S26. Similar to Figure S19, but for both NBE and its first subsequent pulse for event 8.

ID 8

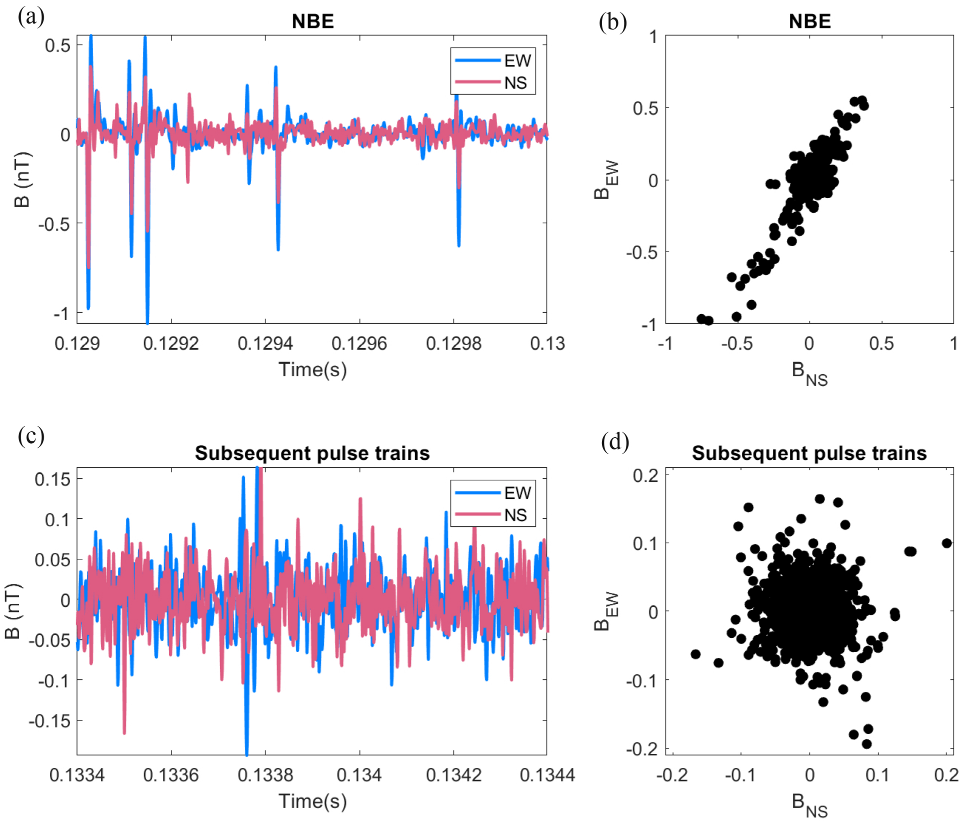

Figure S27. Similar to Figure S19, but for both NBE and its second subsequent pulse for event 8.

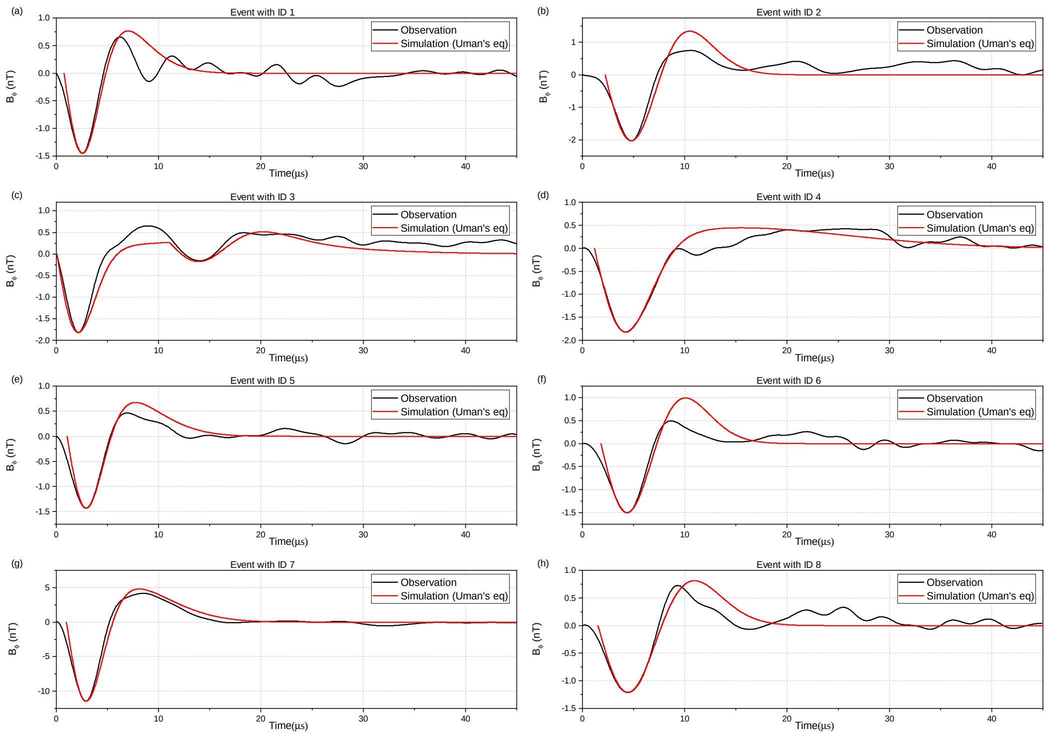

Figure S28. Comparison between the simulation (red) and observation (black) for all the multiple-pulse BLUE cases. The simulation is based on the Uman's equation (Uman et al., 1975).

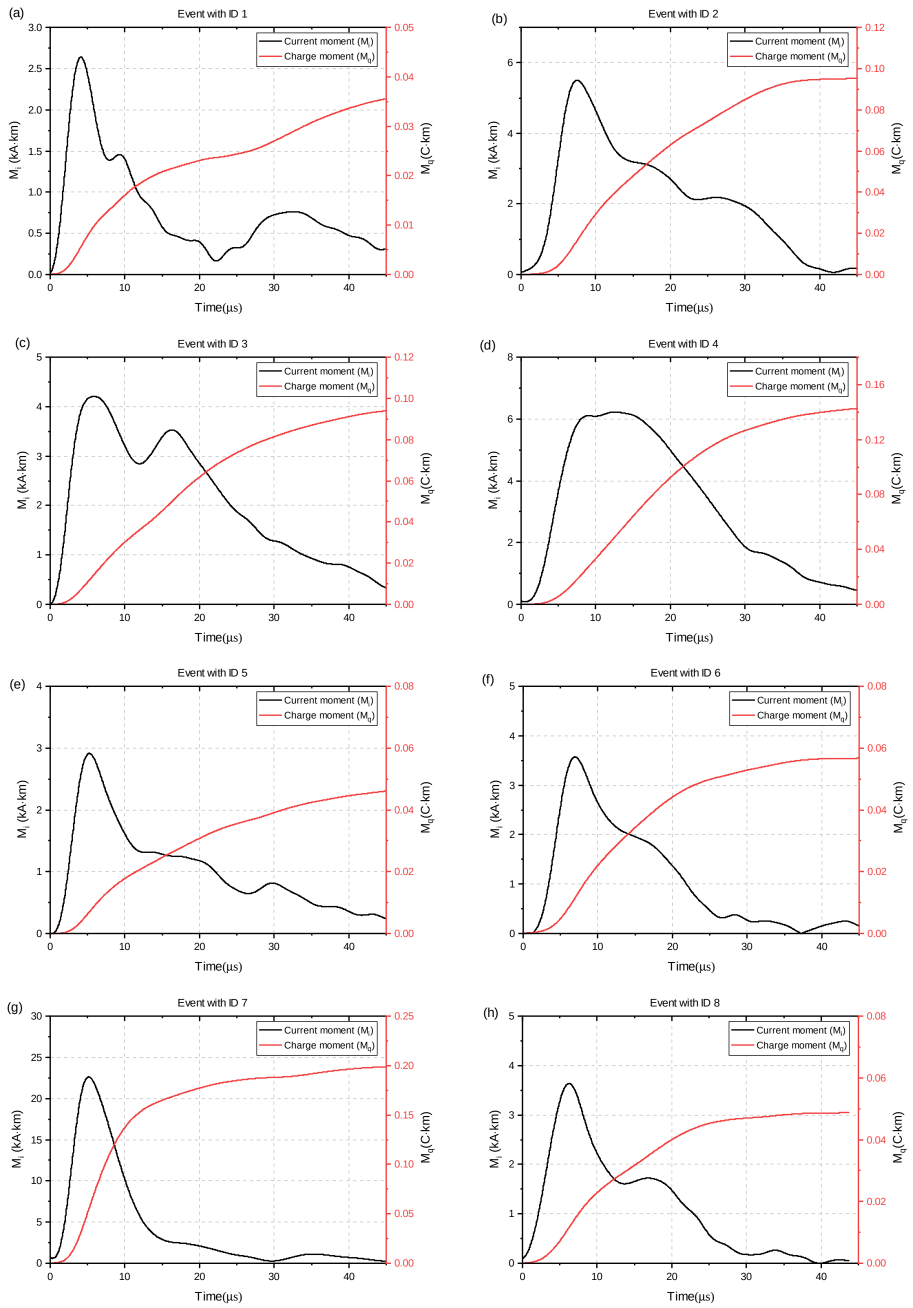

Figure S29. The evaluated current moments M_i and charge moments M_q for the primary BLUE pulse of eight multiple-pulse BLUEs listed in Table 1 based on the azimuthal magnetic field component B_ϕ measured by the ground-based very low frequency/low frequency (VLF/LF) sensor nearby Malaysia.

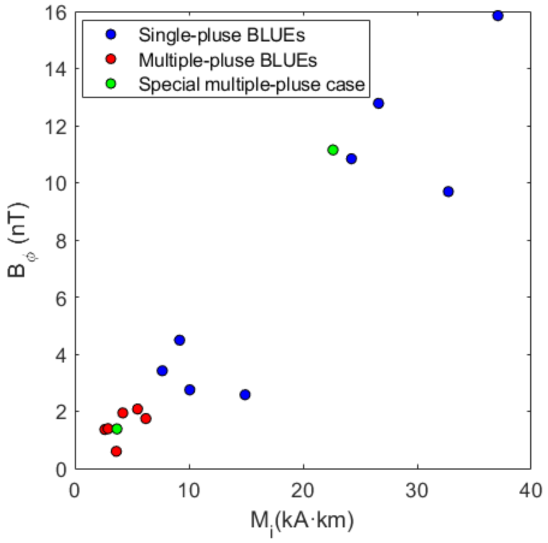

Figure S30. Correlation between the amplitude of the azimuthal magnetic field component B_ϕ and the inferred current moment M_i for all the detected BLUEs (8 single-pulse BLUEs (blue dots), 6 multiple-pulse BLUEs analyzed in the paper (red dots) and 2 special multiple-pulse cases (green dots)). The special cases correspond to the event with ID 6 and ID 7 in Figure S24 and S25, respectively. The current moments of the multiple-pulse events cluster at lower values than those of the single-pulse events. In our set of 16 events, the 7 lowest current moments correspond to multiple-pulse events with the statistical significance of p-value $p = 7 \times 10^{-4*}$.

*To quantify the statistical significance of this fact we must compute the probability that, assuming the same distribution of current moments for multiple and single-pulse events (null hypothesis), we obtain such biased data. Under the null hypothesis all orderings of m multiple pulses and n single pulses are equally probable. There are $\binom{m+n}{n}$ such orderings and out of them, there are $\binom{m+n-s}{n}$ orderings where s multiple-pulse events have the lowest amplitude, where $\binom{\cdot}{\cdot}$ denotes a binomial coefficient. The probability we seek is therefore

$$p = \binom{m+n-s}{n} \times \binom{m+n}{n}^{-1}.$$

In our case with $m = 8$, $n = 8$, $s = 7$ we obtain $p = 7 \times 10^{-4}$, which is small enough to reject the null hypothesis with confidence.

References

Alken, P., Thébault, E., Beggan, C. D., Amit, H., Aubert, J., Baerenzung, J., ... others (2021). International geomagnetic reference field: the thirteenth generation. *Earth, Planets and Space*, *73*(1), 1–25. doi: 10.1186/s40623-020-01288-x

Bilitza, D., Altadill, D., Zhang, Y., Mertens, C., Truhlik, V., Richards, P., ... Reinisch, B. (2014). The international reference ionosphere 2012–a model of international collaboration. *Journal of Space Weather and Space Climate*, *4*, A07. doi: 10.1051/swsc/2014004

Cummer, S. A. (2003). Current moment in sprite-producing lightning. *Journal of Atmospheric and Solar-Terrestrial Physics*, *65*(5), 499-508. (Sprites, Elves and their Global Activities) doi: https://doi.org/10.1016/S1364-6826(02)00318-8

Cummer, S. A., & Inan, U. S. (2000). Modeling elf radio atmospheric propagation and extracting lightning currents from elf observations. *Radio Science*, *35*(2), 385-394. doi: https://doi.org/10.1029/1999RS002184

Koshak, W. J., Solakiewicz, R. J., Phanord, D. D., & Blakeslee, R. J. (1994). Diffusion model for lightning radiative transfer. *Journal of Geophysical Research: Atmospheres*, *99*(D7), 14361-14371. doi: https://doi.org/10.1029/94JD00022

Lehtinen, N. G., & Inan, U. S. (2008). Radiation of elf/vlf waves by harmonically varying currents into a stratified ionosphere with application to radiation by a modulated electrojet. *Journal of Geophysical Research: Space Physics*, *113*(A6), A06301. doi: https://doi.org/10.1029/2007JA012911

Lehtinen, N. G., & Inan, U. S. (2009). Full-wave modeling of transionospheric propagation of vlf waves. *Geophysical Research Letters*, *36*(3), L03104. doi: https://doi.org/10.1029/2008GL036535

Li, D., Liu, F., Pérez-Invernón, F. J., Lu, G., Qin, Z., Zhu, B., & Luque, A. (2020). On the accuracy

of ray-theory methods to determine the altitudes of intracloud electric discharges and ionospheric reflections: Application to narrow bipolar events. *Journal of Geophysical Research: Atmospheres*, *125*(9), e2019JD032099. doi: https://doi.org/10.1029/2019JD032099

Luque, A., Gordillo-Vázquez, F. J., Li, D., Malagón-Romero, A., Pérez-Invernón, F. J., Schmalzried, A., … Østgaard, N. (2020). Modeling lightning observations from space-based platforms (Cloud-scat.jl 1.0). *Geoscientific Model Development*, *13*(11), 5549–5566. doi: https://doi.org/10.5194/gmd-13-5549-2020

Soler, S., Pérez-Invernón, F. J., Gordillo-Vázquez, F. J., Luque, A., Li, D., Malagón-Romero, A., … Østgaard, N. (2020). Blue optical observations of narrow bipolar events by ASIM suggest corona streamer activity in thunderstorms. *Journal of Geophysical Research: Atmospheres*, *125*(16), e2020JD032708. doi: 10.1029/2020JD032708

Uman, M. A., McLain, D. K., & Krider, E. P. (1975). The electromagnetic radiation from a finite antenna. *American Journal of Physics*, *43*(1), 33-38. doi: 10.1119/1.10027

Wilkman, O. (2013). *MieScatter,*. `https://github.com/dronir/MieScatter.jl`. ([Online; accessed 15 Jun 2020])
